# IRESpy: an XGBoost model for prediction of internal ribosome entry sites

**DOI:** 10.1186/s12859-019-2999-7

**Published:** 2019-07-30

**Authors:** Junhui Wang, Michael Gribskov

**Affiliations:** Biological Sciences Department, Purdue University, West Lafayette, IN USA

**Keywords:** Internal ribosome entry site (IRES), Bioinformatics, Machine learning, XGBoost

## Abstract

**Background:**

Internal ribosome entry sites (IRES) are segments of mRNA found in untranslated regions that can recruit the ribosome and initiate translation independently of the 5′ cap-dependent translation initiation mechanism. IRES usually function when 5′ cap-dependent translation initiation has been blocked or repressed. They have been widely found to play important roles in viral infections and cellular processes. However, a limited number of confirmed IRES have been reported due to the requirement for highly labor intensive, slow, and low efficiency laboratory experiments. Bioinformatics tools have been developed, but there is no reliable online tool.

**Results:**

This paper systematically examines the features that can distinguish IRES from non-IRES sequences. Sequence features such as kmer words, structural features such as Q_MFE_, and sequence/structure hybrid features are evaluated as possible discriminators. They are incorporated into an IRES classifier based on XGBoost. The XGBoost model performs better than previous classifiers, with higher accuracy and much shorter computational time. The number of features in the model has been greatly reduced, compared to previous predictors, by including global kmer and structural features. The contributions of model features are well explained by LIME and SHapley Additive exPlanations. The trained XGBoost model has been implemented as a bioinformatics tool for IRES prediction, IRESpy (https://irespy.shinyapps.io/IRESpy/), which has been applied to scan the human 5′ UTR and find novel IRES segments.

**Conclusions:**

IRESpy is a fast, reliable, high-throughput IRES online prediction tool. It provides a publicly available tool for all IRES researchers, and can be used in other genomics applications such as gene annotation and analysis of differential gene expression.

**Electronic supplementary material:**

The online version of this article (10.1186/s12859-019-2999-7) contains supplementary material, which is available to authorized users.

## Background

Internal ribosome entry sites (IRES) are segments of the mRNA, found in untranslated regions, that can recruit the ribosome and initiate translation, especially when the conventional cap-dependent translation initiation mechanism has been blocked or repressed. They have been found to play important roles in viral infection, cellular apoptosis, cellular differentiation and response to external stimuli such as hypoxia, serum deprivation and heat shock [14, 19, 39, 40]. IRES have been identified as potential therapeutic targets for antagonists that can interrupt IRES function and control the expression of viral proteins [23]. Such drugs could be small-molecule inhibitors such as peptide nucleic acids (PNAs), short hairpin RNAs (shRNAs), small interfering RNAs, antisense oligonucleotides, and ribozymes [23, 30, 35]. An improved understanding of cellular IRES function under different physiological conditions will increase our understanding of the response of cells in proliferation, apoptosis and tumorigenesis.

IRES are widely found in both viral and cellular mRNA. They were first discovered in the Poliovirus (PV) and Encephalomyocarditis virus (EMCV) RNA genomes in 1988 using a synthetic bicistronic assay [36]. The assay places potential IRES sequence segments between two reporter genes, and measures the expression of the reporter genes in comparison to a non-IRES control construct. The bicistronic assay is considered to be the best experimental method to confirm the presence of IRES. However, this method is time consuming and labor intensive, and in the past 30 years, only a few hundred IRES have been confirmed. The difficulty of identifying IRES is complicated by our incomplete understanding of the mechanism(s) of IRES function. In the simplest case, that of Dicistroviruses such as cricket paralysis virus (CrPV), IRES function without the help of eukaryotic initiation factors (eIFs) or IRES trans-acting factors (ITAFs), but in other viruses, and in most cellular IRES, eIFs and ITAFs are required. Various lines of evidence implicate RNA structure in IRES function [7, 26, 31, 37], especially in IRES that do not require additional protein factors, but the relative importance of RNA structure, ITAFs, and (possibly unidentified) RNA binding proteins remains unclear. Whether all IRES share a common mechanism, and therefore common sequence and structural features, has not been determined, and universal features shared by all IRES have yet to be identified [22, 28]. This substantial gap in our knowledge can be largely attributed to the relatively small number of confirmed IRES, which has made identification of common features difficult.

It has been estimated that about 10% of cellular and viral mRNA may use IRES to initiate translation [41], but the limited number of confirmed IRES has prevented study and understanding of IRES function. Alternative approaches to IRES identification, such as comparative analysis of IRES primary/secondary/tertiary structure, have been tried, but little commonality has been found across all IRES [7, 12]. Small sequence motifs have been reported to be conserved within specific viral IRES groups, for instance, a GNRA sequence is shared in picornavirus IRES [5]. The SL2.1 stem/loop contains a U rich motif that has been found to be important for ribosome binding in the Dicistrovirus intergenic region (IGR) IRES [4, 38].

The absence of universally conserved features across all IRES makes their prediction difficult from a bioinformatics perspective, but several systems have been implemented. For example, the Viral IRES Prediction System (VIPS) predicts the secondary structure of an RNA from its sequence, and uses the RNA Align program to align the predicted structure to known IRES to predict whether the sequence contains an IRES [12]. However, VIPS predictions are limited to viral IRES, and although the accuracy rate of VIPS was assessed as over 80% for four viral IRES sub-groups, the prediction accuracy was assessed only on the training dataset and is substantially overestimated. The ability of VIPS to find novel viral IRES is low in our hands (note that the VIPS server is no longer available). A more recent method, IRESPred, uses 35 sequence and structural features and the probabilities of interactions between RNA and small subunit ribosomal proteins to predict IRES [21]. IRESpred was trained using a non-IRES negative training set that included viral protein coding and cellular protein coding mRNA sequences; unfortunately some of these sequences were later found to contain IRES [46]. In addition, IRESpred incorporates features such as UTR length and the number of upstream AUGs. Such features are dependent on the length of the query sequence, and most of the positive training set is substantially longer than the negative training set. The overall false positive rate for IRES prediction with IRESPred is high: in a test of 100 random 400 base sequences, 98 were predicted to be IRES (results not shown). This high false positive rate has been confirmed by other investigators, as well [50].

Instead of using features common to all IRES to determine for prediction, recent results suggest that machine learning approaches that combine multiple weak learners to predict IRES may be effective [25, 44]. In 2016, Weingarten-Gabbay et al. developed a high-throughput IRES activity assay and employed it to identify thousands of novel IRES in human and viral genomes [46]. The identification of many new IRES improves the likelihood that a machine learning model can be successfully implemented. Based on the Weingarten-Gabbay et al. dataset, Gritsenko et al. built a stochastic gradient-boosting decision tree model (GBDT) [8, 48] to predict IRES using 6120 kmer features [10]. However, the large feature set leads to possible model overfitting and slow model fitting time.

IRESfinder, the most recent method, uses only the human genome part of the Weingarten-Gabbay et al. dataset and implements a logit model with framed kmer features to predict cellular IRES [50]. The IRESfinder logit model was trained only on cellular IRES, and, as a transformed linear model, may not work well for non-linear relationships. In addition, the independent testing dataset is very small (only 13 sequences), possibly leading to overestimation of the AUC.

In this manuscript, we describe a machine learning model that combines sequence and structural features to predict both viral and cellular IRES, with better performance than previous models. In order to make the predictive model widely available, it has been implemented as a simple to execute R/Shiny app. The optimized model, IRESpy, is very fast, and can be used to make genome scale predictions.

## Results

In a typical scenario, one has only the sequence of the RNA available and does not have additional information (such as experimentally determined secondary and tertiary structure). In this work, we focus on features that can be obtained from the sequence alone, rather than on comparative information, which requires a curated comparative database. We consider three kinds of features: sequence features, structural features, and sequence-structural hybrid features.

### Sequence features

**Fig. 1:**
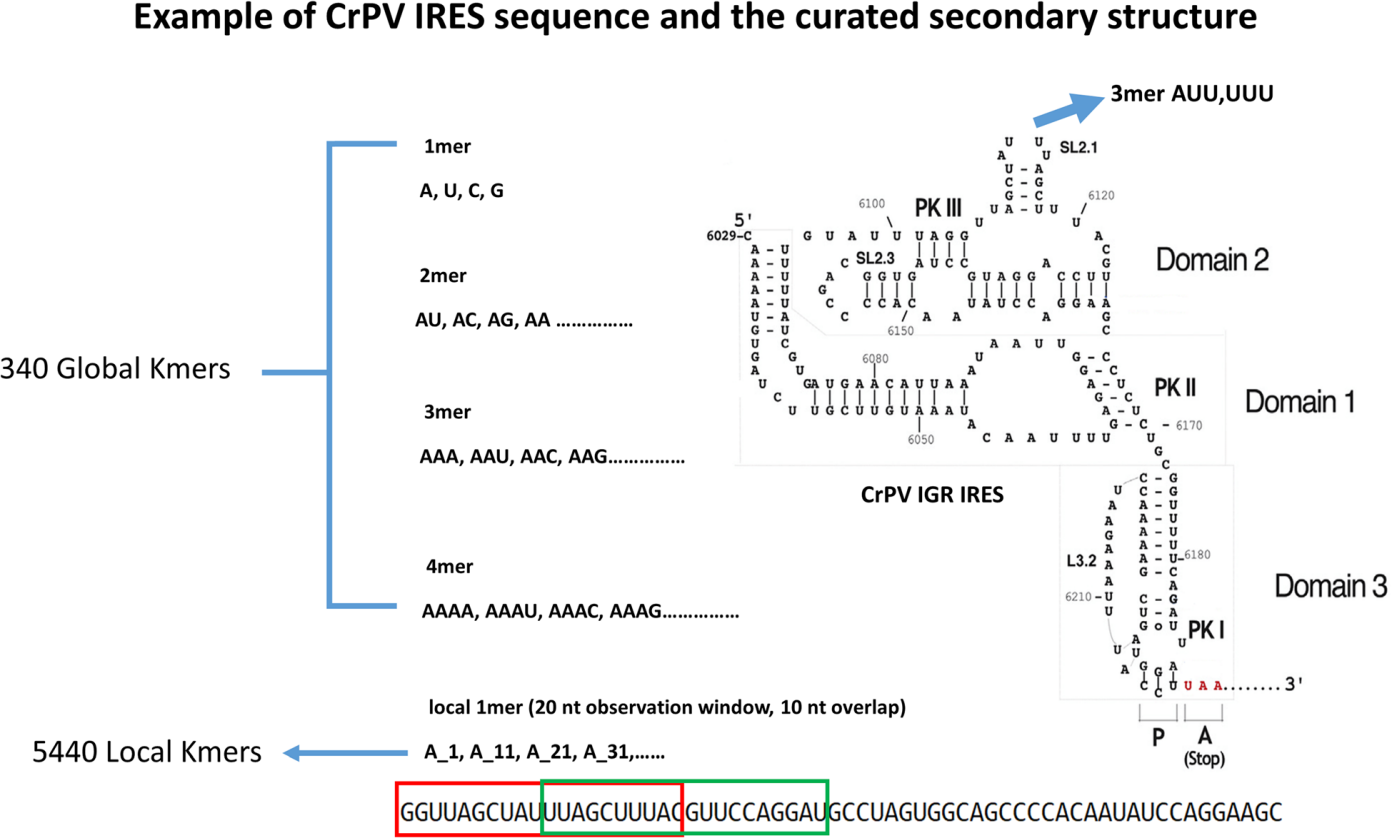
Calculation of Kmer features. An example of kmer features in the Cricket paralysis virus (CrPV) intergenic region (IGR) are shown. From 1mer to 4mer examples are shown. The red and green boxes show examples of the observation window used to calculate local kmers. 340 global kmers and 5440 local kmers have been tested in this research

Sequence features are the tabulated frequencies of kmer words in the target sequences. Given the four base RNA alphabets, there are 4^*k*^ words of length *k*, yielding four 1mer, sixteen 2mer, sixty-four 3mer, and two hundred and fifty-six 4mer features (total = 340). It is possible that sequence features, which might correspond to protein binding sites, could be localized with respect to other features in the IRES. To incorporate this possibility, we consider both global kmers, the word frequency counted over the entire length of the sequence, and local kmers, which are counted in 20 base windows with a 10-base overlap, beginning at the 5′ end of the sequence of interest. In all cases, the kmer count is divided by the sequence length to give the kmer frequency. An example of kmer calculation for the Cricket Paralysis Virus intergenic region (CrPV IGR) IRES is shown in Fig. 1.

### Structural features

The predicted minimum free energy (PMFE) is highly correlated with sequence length [42]. This is undesirable as could lead to false positive predictions based on the length of the query sequence. While this effect is reduced using Dataset 2, in which all training sequences are the same length, sequence length is clearly a conflating variable that should be excluded.

Q_MFE_, the ratio of the PMFE and the PMFE of randomized sequences [1], is much less dependent on sequence length (see methods). It is believed that the stability of RNA secondary structure depends crucially on the stacking of adjacent base pairs [15, 43]. Therefore, the frequencies of dinucleotides in the randomized sequences are an important consideration in calculating the PMFE of randomized sequences [3]. In calculating Q_MFE_, a dinucleotide preserving randomization method has been used to generate randomized sequences.

Q_MFE_ can be used to compare the degree of predicted secondary structure in different sequences regardless of length. This length independent statistic indicates whether the degree of secondary structure is relatively lower or higher than that of randomized sequences, respectively. Viral IRES have been found to have highly folded secondary structures that are critical for their function. The structures of Dicistrovirus IRES, in particular, are conserved and comprise folded structures with three pseudoknots. Cellular IRES typically need ITAFs to initiate translation, and the binding between ITAFs and cellular IRES has been proposed to activate the IRES structure by changing it from a relaxed status to a rigid status [7]. Cellular IRES are therefore likely to have a less extensively base-paired secondary structure. The 5′ UTRs of housekeeping genes, in general, do not require highly folded structures because they use the cap-dependent translation initiation process.

**Fig. 2:**
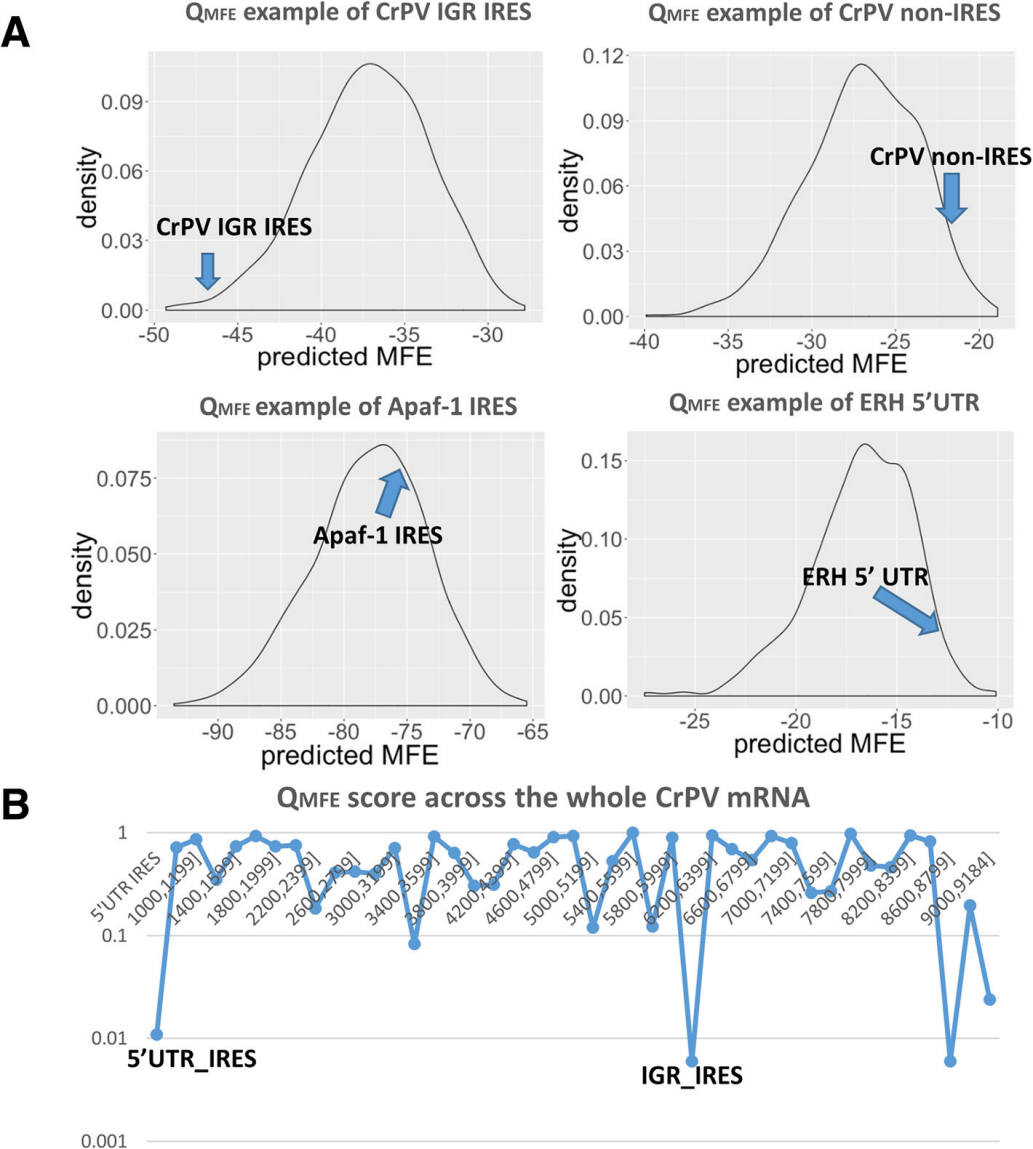
Q_MFE_ calculation examples of IRES and non-IRES sequences. **a** PMFE of randomized sequences (density plot) and PMFE of the CrPV IGR IRES (viral IRES, PMFE = -47.5, Q_MFE_ = 0.001), the ERH 5′ UTR (housekeeping gene, PMFE = -12.7, Q_MFE_ = 0.99), Apaf-1 cellular IRES (PMFE = -76, Q_MFE_ = 0.66), and CrPV non-IRES regions (position: 6200–6399, PMFE = -22.2, Q_MFE_ = 0.94). **b** Q_MFE_ of 200 base segments across the whole genomic CrPV mRNA. The Q_MFE_ shows minimal values in the regions of the known the 5’UTR IRES (bases 1–708) and IGR IRES (bases 6000–6200)

Average Q_MFE_ values clearly differ in viral IRES, cellular IRES and the UTRs of housekeeping genes (Fig. 2). We expect that Q_MFE_ also should be different in IRES and non-IRES regions of the same mRNA. Figure 2a shows the observed differences in Q_MFE_ of selected viral IRES, cellular IRES, and a housekeeping gene 5’UTR. The Q_MFE_ of the viral IRES is the lowest, indicating the presence of a more stable folded structure. The cellular IRES Q_MFE_ is about 0.5, which indicates this sequence has an intermediate degree of secondary structure, but still more than would be expected for randomized sequences, and the 5’UTR of the ERH housekeeping genes has the highest Q_MFE_, indicating a relatively low degree of secondary structure. These results suggest that the Q_MFE_ can indicate the degree of base-paired secondary structure in various sequence classes, and may be useful in distinguishing IRES and non-IRES sequences. Figure 2b shows the Q_MFE_ of 200 base segments of CrPV. Two of the low Q_MFE_ regions exactly match the regions of the known the 5’UTR IRES (bases 1–708) and intergenic (IGR) IRES (bases 6000–6200), again indicating that Q_MFE_ may be a powerful discriminatory feature that can be used to identify IRES positions mRNA sequences.

### Hybrid features

**Fig. 3:**
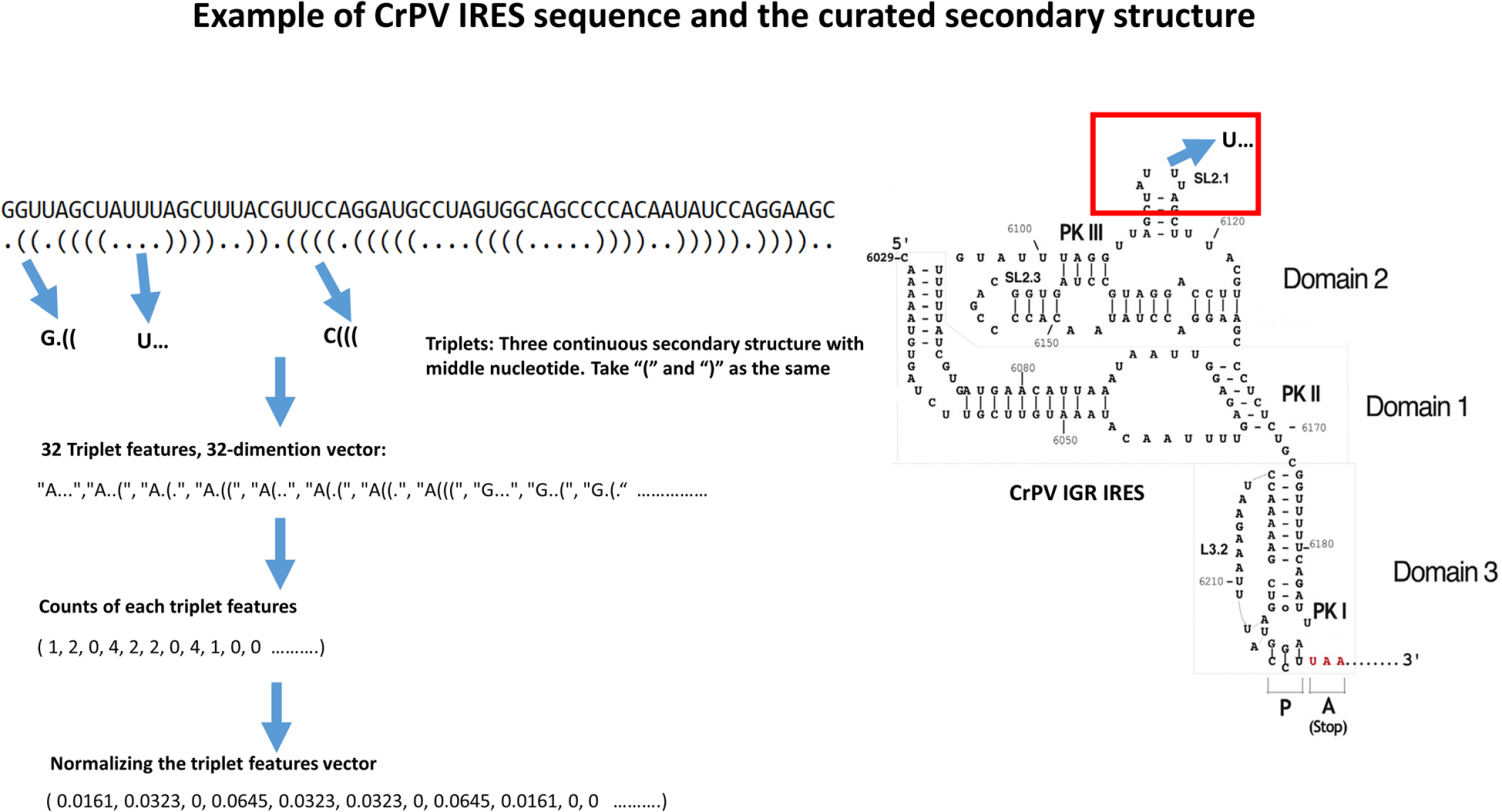
Calculation of triplet features. An example of triplet features in the Cricket paralysis virus (CrPV) intergenic region (IGR) are shown. The secondary structure of the candidate sequence was predicted using UNAfold [29]. For each nucleotide, only two states are possible, paired or unpaired. Parenthesess “()” or dots “.” represent the paired and unpaired nucleotides in the predicted secondary structure, respectively. For any 3 adjacent bases, there are 8 possible structural states: “(((”, “((.”, “(..”,“(.(”,“.((”,“.(.”,“..(”, and” …”. Triplet features comprise the structural states plus the identity of the central base, A, C, G, or U, so there are 32 (8*4 = 32) triplet features in total. Triplet features are normalized by dividing the observed number of each triplet by the total number of all the triplet features

Triplet features, which combine the primary sequence and predicted base-paired structure, have been used in miRNA prediction [45]. The first successful application of this kind of feature was in a support vector machine algorithm for classifying pre-miRNAs [47]. The definition and calculation of triplet features are shown in Fig. 3. Triplet features encode the local predicted secondary structure as a series of characters indicating the predicted structure (where the symbols ‘(‘ and ‘.’ indicate base-paired and unpaired bases, respectively) and the base at the center of the triplet. The triplet feature “A((( “thus indicates a sequence where three bases are base-paired, and the center base is an ‘A’.

### Approach

In this work, we focus on an ab initio classification approach for IRES prediction. All the features considered here are sequence length independent - kmer words, Q_MFE,_ and triplets, and thus should be equally appropriate for scanning long (genomic) or short (specific target) sequences.

Two existing databases have been created to systematically study IRES, which provide useful background information for this study. The first database, referred to as Dataset 1 in this work, comprises confirmed IRES drawn from IRESite [33] and includes selected 5’UTRs of housekeeping genes. Fifty-two viral IRES and 64 cellular IRES from IRESite are labeled as IRES in Dataset 1. Housekeeping genes principally utilize the 5′ cap-dependent mechanism for initiation, and 51 of them were randomly selected as the non-IRES group used for comparison in Dataset 1 [24]. Dataset 2 is derived from a high-throughput bicistronic assay that has increased the number of known IRES by more than 10-fold [46]. This large increase in the number of examples of IRES provides an opportunity to better learn the relationship between sequence and structural features and IRES mechanism. We primarily rely on the Dataset 2 to build the machine learning model due to its large size and semi-quantitative measure of IRES activity. Dataset 2 only contains only human and viral IRES, and all sequences share the same length. To explore all other IRES from other species and with various lengths, and to provide an independent test set, Dataset 1 is used.

Dataset 2 has been randomly divided into a training partition (90%) and a validation partition (10%). The training dataset was used in a grid search to optimize the XGBoost model parameter: learning rate, maximum tree depth, subsample ratio of the training instances, and subsample ratio of the features, used when constructing each tree (Additional file 1: Figure S3). Each combination of parameters was evaluated using 10-fold cross validation, in which the training partition is equally divided into 10 sets; one set is used for testing, and the remainder used for training in each run. In successive runs, different partitions are held out for testing. In the end, the best fit parameters are summarized to generate the final set of model parameters. The data in the validation is not included in either hyperparameter or parameter training and thus provides an unbiased evaluation of the final trained model. The whole nested cross validation process is described in detail in section 1 of the Additional file 1.

XGBoost stands for eXtreme Gradient Boosting. It combines weak learners (decision trees) to achieve stronger overall class discrimination [2]. XGBoost learns a series of decision trees to classify the labelled training data. Each decision comprises a series of rules that semi-optimally splits the training data. Successive trees that “correct” the errors in the initial tree are then learned to improve the classification of positive and negative training examples. Compared with gradient boosting, XGBoost can be more efficiently parallelized, and incorporates regularization and tree pruning to reduce over-fitting. A variety of hyperparameters must be optimized in the XGBoost method, including the learning rate, maximum tree depth, subsample ratio of the training instances, and subsample ratio of the features.

A succession of decision trees are generated where each tree, metaphorically, corrects the errors made in the previous trees. Due to the nature of this process, it is often difficult to map the importance of the features directly onto biological importance since each individual “rule” in the decision tree is likely to be noisy.

### Training on kmer features

**Fig. 4:**
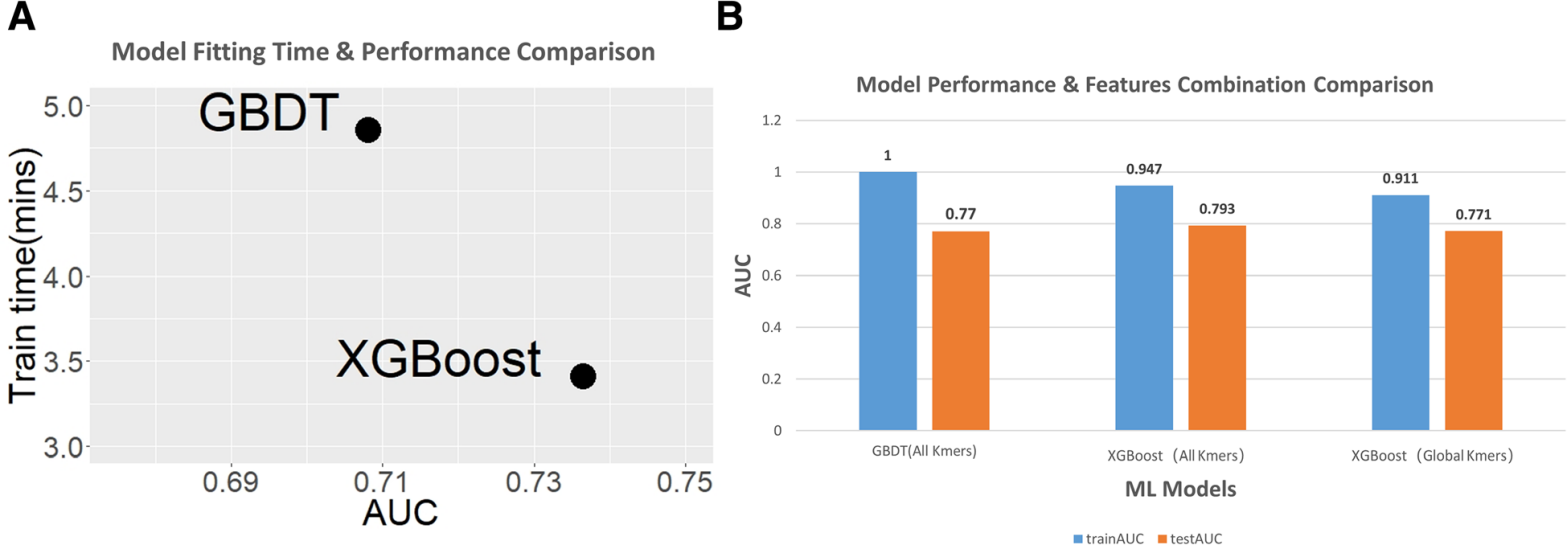
Model performance of XGBoost and GBDT. **a** The model performance of XGBoost and GBDT for only the global kmer features, without any hyperparameter tuning. **b** Model performance comparison using area under the ROC curve (AUC). The XGBoost model has lower training AUC but higher testing AUC than the GBDT model. The XGBoost model trained with only local kmers performs the same as the GBDT model, but the number of features is reduced from 5780 to 340

Machine learning models, including GBDT, and extreme gradient boosting (XGBoost), have been compared for IRES prediction. The approach used here, XGBoost exhibits higher AUC performance, and substantially lower training time than the GBDT model. As shown in Fig. 4a, XGBoost requires 75% less training time, but improves AUC by 5% compared with GBDT, without any hyperparameter tuning. With the same features, but different model and parameter tuning, the XGBoost model can reach a testing AUC of 0.793 and training AUC 0.947. This is substantially better than the GBDT which showed a testing AUC of 0.77, and training AUC of 1.0 (Fig. 4b). To investigate the relative importance of global and local kmer features, the XGBoost model was run with the same parameter settings, but incorporating only global kmer features. In this case, the testing AUC is 0.771 and training AUC is 0.911 (Fig. 4b); this model achieves the same performance as GBDT, but requires many fewer features. The final model includes 1281 individual trees and each tree incorporates 340 features. The maximum depth of each tree is set to be 6.

### Training on kmer + structural features

**Fig. 5:**
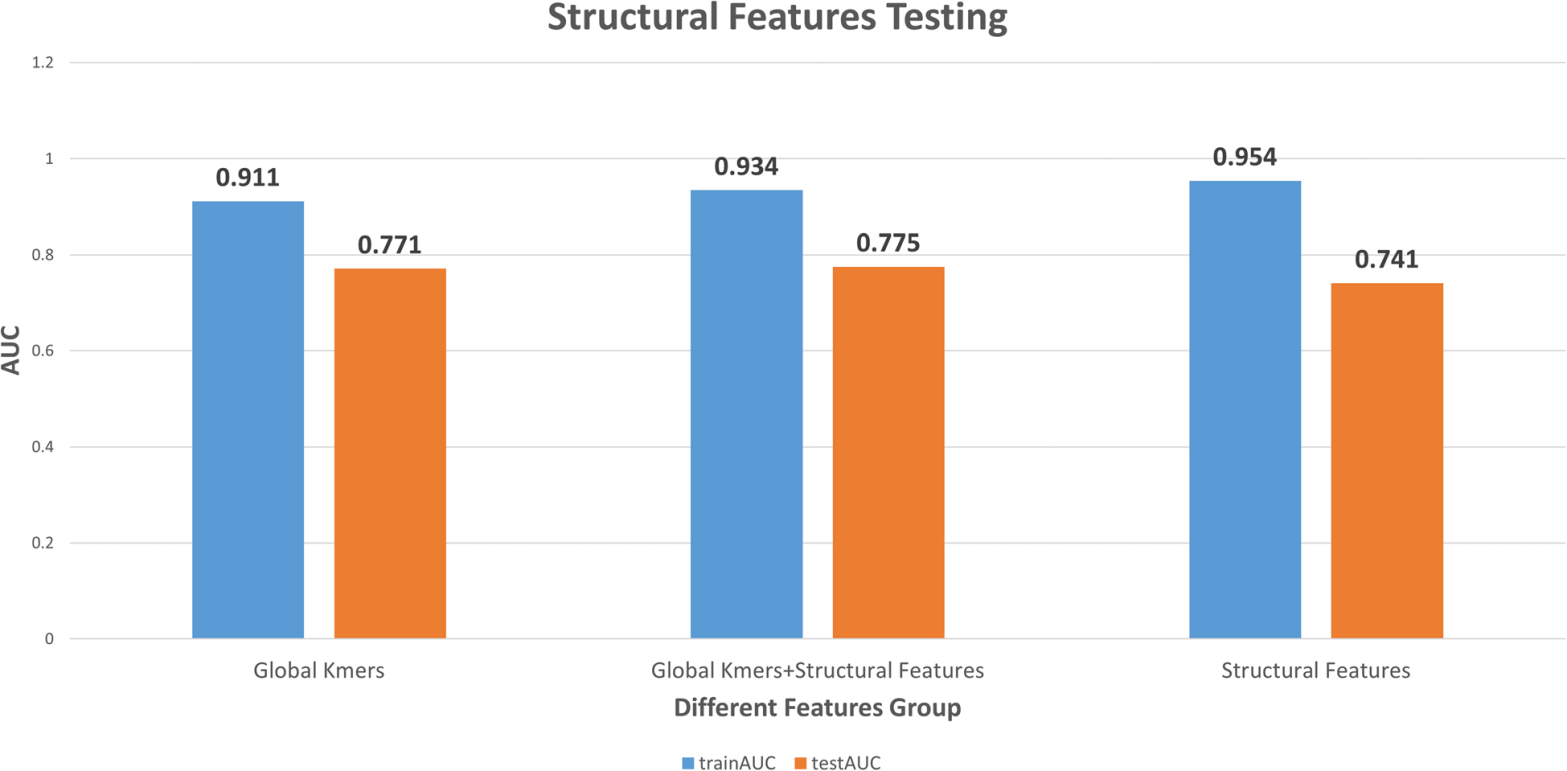
Effect of incorporating structural features. QMFE and triplet features were included in a combined model with global kmer features. We examined models incorporating only global kmer features, only structural features, and a combination of global kmer and structural features

Structural features such as the number of predicted hairpin-, bulge-, and internal- loops; maximum loop length, maximum hairpin-loop length, maximum hairpin-stem length, and the number of unpaired bases have been previously studied [10, 21, 50], but none were found to have significant predictive value. We hypothesized that Q_MFE_, and triplet features, because they are length independent and combine sequence and structural information, might act as better features to classify IRES and non-IRES sequences. In particular, triplet features have the potential to reveal locally conserved sequence motifs that appear in a specific structural context. These features have been combined with the previously examined global kmer features in a sequence-structural model that is better than the simple sequence-based model. The testing AUC of the combined model increases slightly, from 0.771 to 0.775 (Fig. 5). The small magnitude of the increase probably indicates the presence of correlation between the global kmer and structural features. When using the structural features alone, the testing AUC is 0.741, which means that the structural features can still capture most of the variance of the dataset with only 33 features.

The high AUC of the structural feature-based model indicates that structural features alone can capture most of the information contained in the kmer features, while decreasing the number of features from 340 to 33. The structural features therefore have a relatively high information content. However, the lack of improvement in the combined model compared to either the global kmer or structural model suggests that the information in kmer words and the structural features may be largely redundant.

### Biological significance of discriminative features

**Fig. 6:**
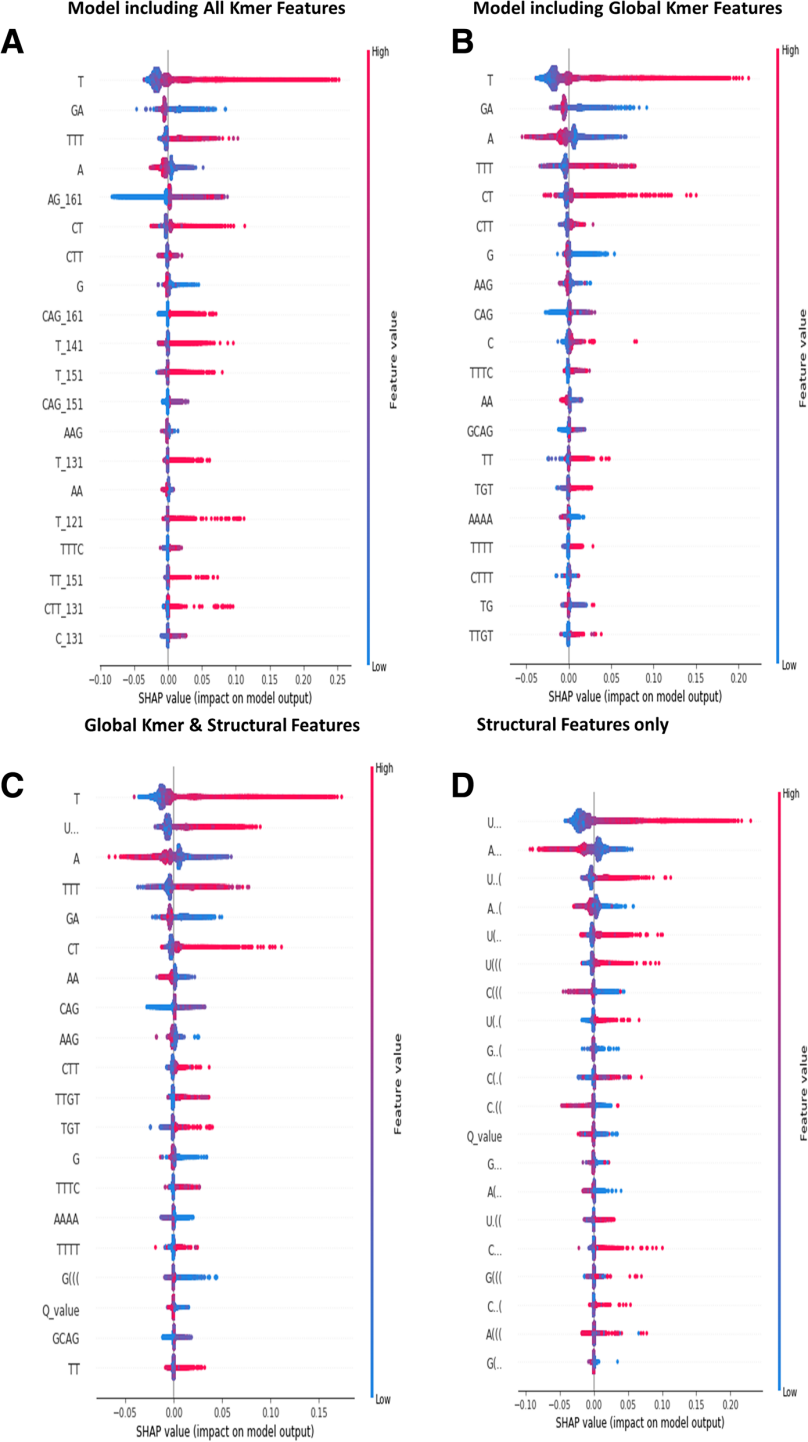
XGBoost model feature importance explained by SHAP values at the global scale. **a** The summary of SHAP values of the top 20 important features for model including both global kmers and local kmers. **b** The summary of SHAP values of the top 20 important features for models including only global kmers. **c** The summary of SHAP values of the top 20 important features for models including both global kmers and structural features. **d** The summary of SHAP value of the top 20 important features for model including only structural features

As mentioned previously, it is not usually straightforward to understand the biological relevance of the selected features. Machine learning (ML) models are often considered “black boxes” due to their complex inner mechanism. Understanding the contribution of each feature to the model has been recognized as a very difficult aspect of machine learning. The SHAP (SHapley Additive exPlanations) method assigns values that measure the marginal contribution of each feature to the model [27]. It combines game theory with local explanations and is well suited for machine learning explanation. Unlike feature importance measures based on weight, cover, or information gain, the SHAP value is the only consistent and locally accurate additive method, and it can be interpreted as indicating which features are the most globally important for classification. Figure 6a shows the top 20 most important features in models trained with both global and local kmers. Red indicates higher feature values and blue indicates lower feature values. Higher frequencies of U rich kmers, such as “U”, “UU”, “UUU”, “UUUU”, “CU”, and “UGU”, are associated with higher predicted probability of being IRES. This is consistent with the previous reports that pyrimidine-rich kmers, especially U rich kmers are important for IRES function [46]. Importance of global kmer and local kmer features follow similar patterns, for instance, the local kmer features U_121, U_131, U_141, U_151, and U_161 all support classification of sequences as IRES, as do the global kmer features. The importance of the local region from base 121–161 may be important as an ITAF binding site (perhaps pyrimidine tract binding protein), as suggested by Weingarten-Gabbay et al. Whether the CU feature is related to the poly U feature is difficult to tell. It is worth noting that in picornaviral IRES, one of the most conserved features is the SL3A “hexaloop” in which a CU dinucleotide is highly conserved [6]. Figure 6b lists the SHAP values of the top important features for the global kmer only model. The similar importance of features in different models suggests that the models are detecting essentially the same features. Figure 6c shows the SHAP values for both the global kmer and structural features model. Some structural features, such as ‘U..’, ‘G(((’, and the Q_MFE_, are more important than most global kmers. Figure 6d lists the structural features, and serves as a potential structural motif list much like a differentially expressed genes list in the RNA-seq analysis.

**Fig. 7:**
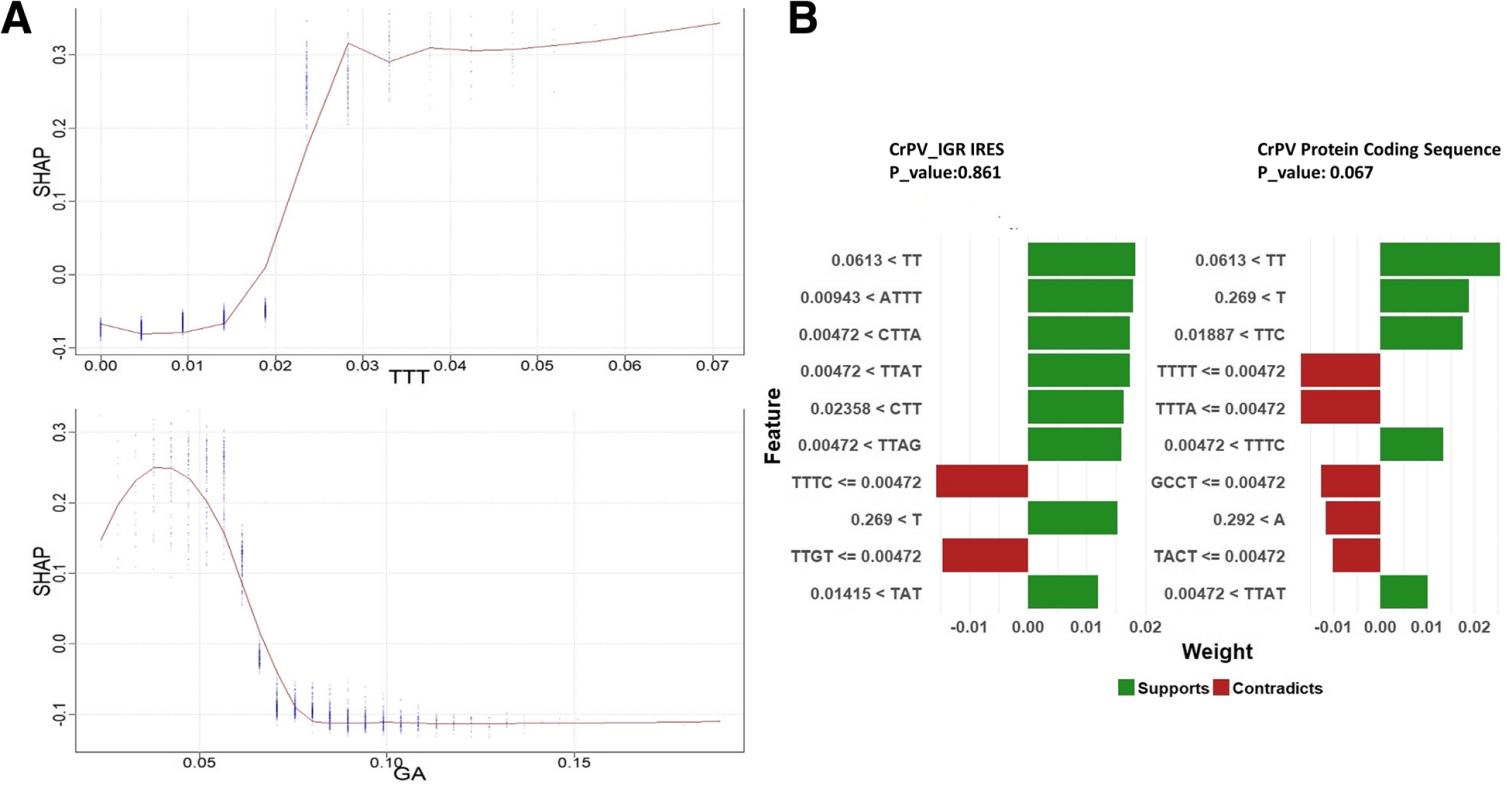
XGBoost model feature importance explained by SHAP and LIME at a local scale. **a** SHAP (SHapley Additive exPlanation) dependence plots of the importance of the UUU and GA kmers in the XGBoost model. **b** Local Interpretable Model-agnostic Explanations (LIME) for the CrPV IGR IRES and CrPV protein coding sequence. The green bar shows the weighted features that support classification as IRES and red bars are the weighted features that oppose classification as IRES

In order to understand the biological meaning of the trained model we can examine how the response variable, in this case classification as IRES vs non-IRES, changes with respect to the values of the features. SHAP values show the change in the predicted value as a specified feature varies over its marginal distribution, for each important feature. Figure 7a shows examples of two highly ranked features. An increase in the frequency of the UUU 3mer, from 0.01 to 0.03, increases the probability that a sequence is an IRES, while an increase in the frequency of the GA 2mer from 0.04 to 0.08 decreases the probability that the sequence is IRES.

For novel sequences, instead of simply predicting the probability that a sequence is an IRES, we want to know which features can explain the prediction. Local Interpretable Model-agnostic Explanations (LIME) analysis explains the contribution of individual features to the overall prediction [20, 49]. The assumption of LIME is that every complex model has a linear or explainable relationship in the local space of the dataset. It is possible to fit a simple model around a sequence by slightly permuting its feature matrix. In LIME, a similarity matrix that measures the distance between a query sequence and a certain number of permutations is constructed. Each permutation is classified by the XGBoost model, and the predicted class, IRES or non-IRES, is classified by a simple model. The simple model uses the same features as the XGBoost model, and mimics how the XGBoost model behaves in the local space defined by the permutations. Figure 7b shows, for instance, why the predicted probability of CrPV IGR IRES is high (*p* = 0.861), but the predicted probability of an IRES in the CrPV protein coding sequence is very low (*p* = 0.067). The green bars, which represent the positively weighted features, are more prominent in the CrPV IGR IRES, than in the CrPV protein coding sequences (non-IRES).

We use importance ranking plots to analyze the importance of triplet features in IRES prediction. Figure 6b shows that triplets “U …” , “A …” , “A..(” are important in the model including both global kmers and structural features, as well as in the model including only structural features. In particular, the triplet “U …”, a loop with a central U base, can be seen to be important. This feature may correspond to the conserved U rich loop motif found in the SL2.1 region of Dicistrovirus IGR IRES. The SL2.1 stem/loop has been found to be important for ribosome binding [4, 38], and in the Cryo-EM structure of the CrPV IRES, it is complexed with the ribosome, with the SL2.1 region positioned at the interface of the IRES and the ribosome [16, 38], in direct contact with the ribosome. Mutations in the SL2.1 region result in loss of IRES function [11, 17, 28].

### Prediction probability vs IRES activity

**Fig. 8:**
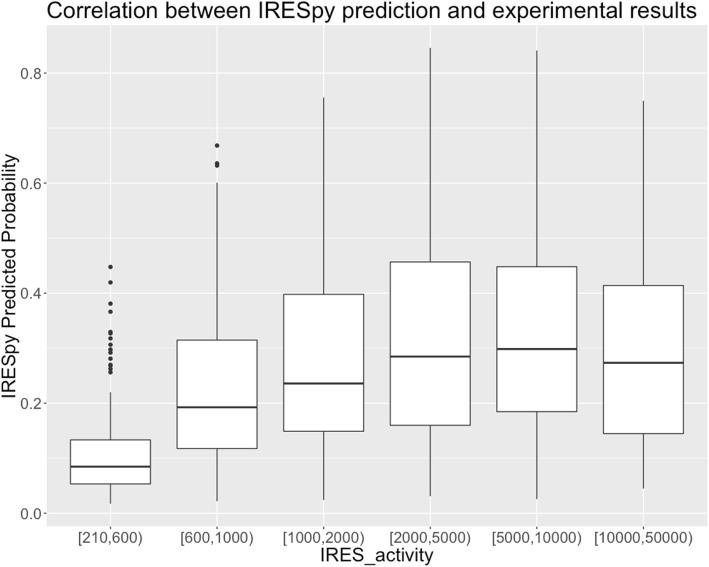
Correlation between IRESpy prediction and experimental results

The IRES activity of the sequences in Dataset 2 was measured by inserting them into a lentiviral bicistronic plasmid, between mRFP and eGFP reporter genes, and transfecting H1299 cells, which results in integration of a single oligonucleotide construct in each cell [46]. The cells were sorted with FACS and assigned to 16 fluorescence intensity bins on the basis of eGFP expression. IRES activity, in the range 206 to 50000, is defined by those expression levels. The correlation between the IRES probability predicted by our XGBoost model and the quantitative IRES experimental activities has been explored, and the result shows that the predicted IRES probability is significantly higher for high-activity (> 600) IRES, than for those where the IRES activity is close to the base level (≤600) in Fig. 8. This suggests that the predictive accuracy of the XGBoost model is higher for high activity IRES than for marginally active sites, and implies that, when high precision is a priority, precision can be increased at the expense of recall.

### Scan of human UTRs

**Fig. 9:**
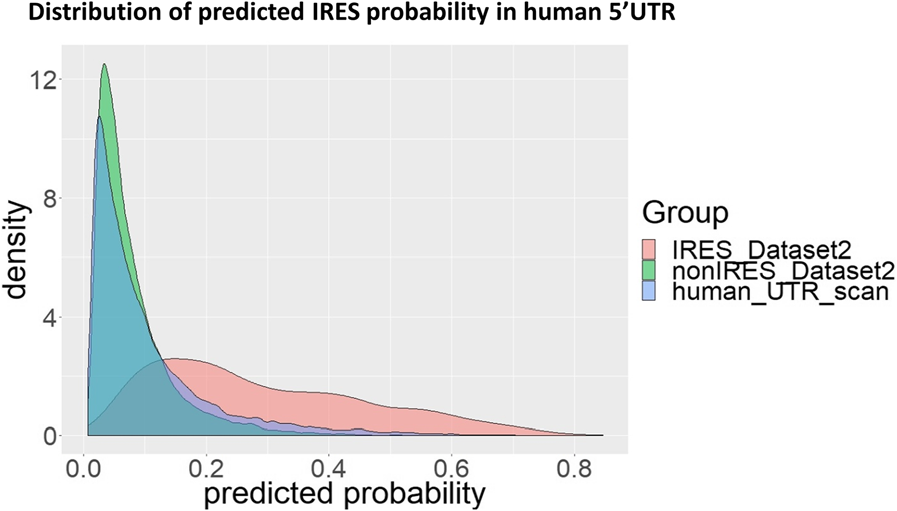
The density distribution of predicted IRES probability in Dataset 2 and human UTR scan

IRESpy has been applied to scan human 5’UTRs (124315 UTR sequences listed in UTRdb). Figure 9 shows the distribution of IRES prediction probability for the positive and negative training sets in Dataset 2, and all human UTRs. The distribution of probabilities in the human UTR dataset strongly resembles the Dataset 2 negative class, but has a larger tail. This suggests that IRESpy is successfully distinguishing IRES from non-IRES in the uncharacterized human UTRs. When a prediction threshold of 0.1 is used for both datasets, 13.47% of the human IRES are predicted to contain IRES which is close to the 10% value cited in previous reports [41].

### IRESpy prediction tool

The XGBoost model based on global kmer features, has been implemented as a shiny application, IRESpy. It is available online: https://irespy.shinyapps.io/IRESpy/. Compared with IRESpred (Table 1), IRESpy shows better predictive performance, with both higher sensitivity (recall) and higher precision on the validation dataset (not included in parameter or hyperparameter training).

**Table 1:**
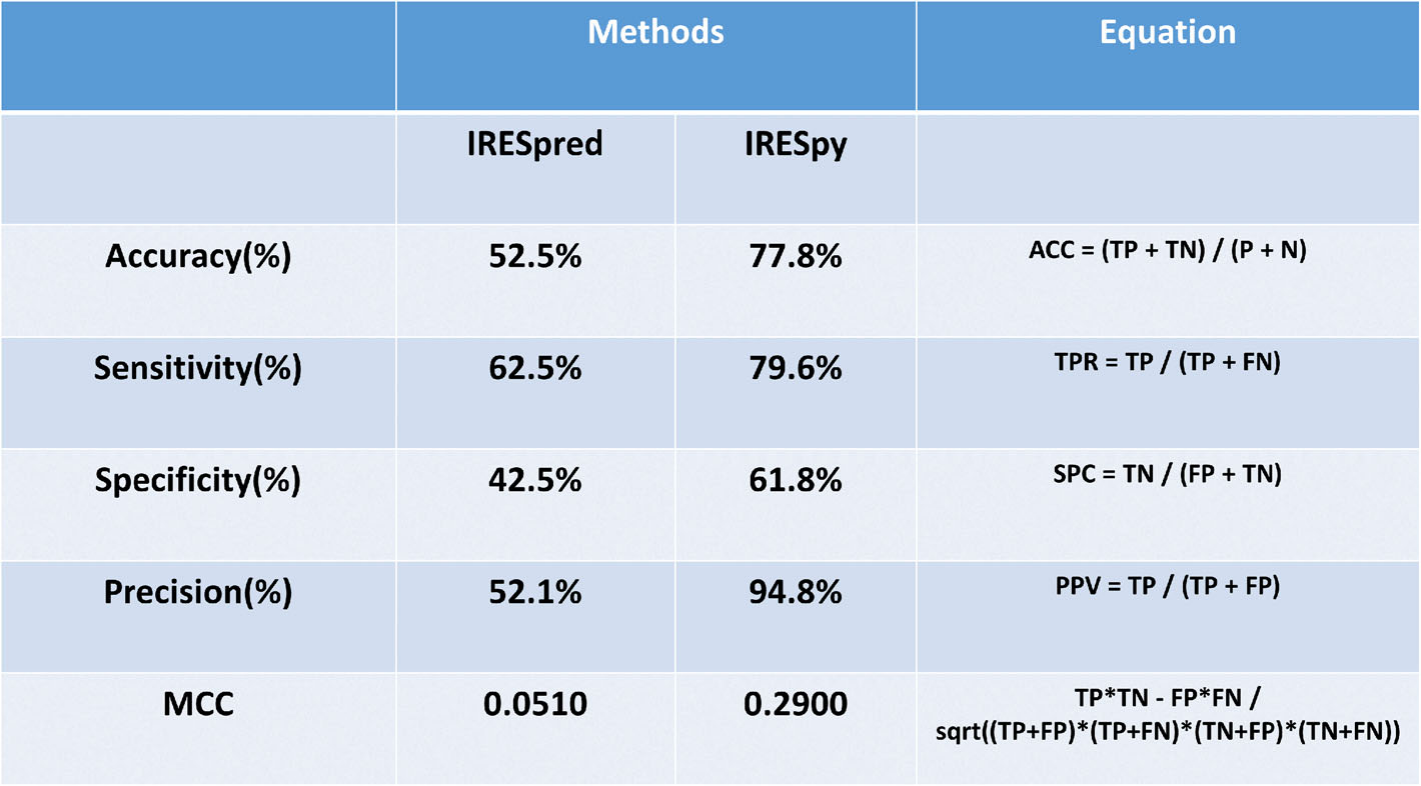
Comparison between IRESpy and IRESpred model performance. IRESpy performs better than IRESpred in accuracy, sensitivity (recall), specificity, precision and MCC

**Fig. 10:**
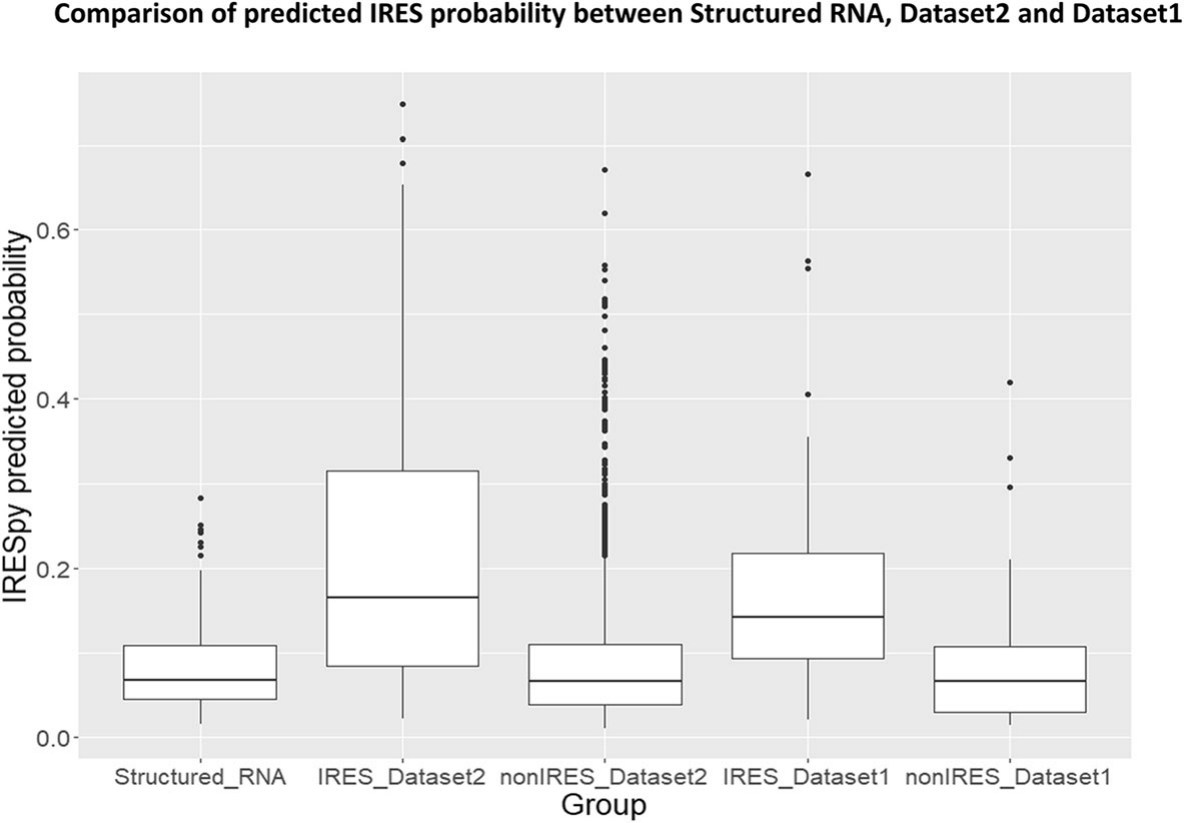
Predicted probability of IRES for highly structured RNA families, and IRES and non-IRES classes in Datasets 1 and 2

To further test the predictive ability of IRESpy, it has been applied to 202 highly structured non-IRES RNAs (see methods) [13], to Dataset 1, which includes the reported sequences of IRES from IRESite (positives) [33], and to housekeeping gene 5’UTRs (presumed negatives). IRESpy clearly distinguishes IRES and non-IRES sequences in Dataset 1. The low predicted IRES probability for all highly structured RNA groups suggests that IRESpy is not simply detecting relatively structured RNA. Since a relatively high amount of secondary structure is widely considered to be a hallmark of IRES, the test against highly structured RNAS represents an especially difficult test (Fig. 10).

## Discussion

Clearly, both the selected features and the models are important for predicting the existence of IRES. A limitation of VIPS and IRESPred are the inclusion of length dependent features such as the length of UTRs, and the number of upstream AUGs. This is a serious drawback when predicting IRES in UTRs, which vary greatly in length. IRESpy performs better than the GBDT method, using a smaller number of features. Using the same datasets and features (global and local kmer features), but switching from the GBDT model to XGBoost, increases the validation AUC by 5%, and the decreases the training time by 75%.

Global kmer and local kmer features are highly correlated. The XGBoost model achieves the same model performance as the GBDT model incorporating only global kmer features. The modest increase in classification performance, accompanied by a 94% decrease in the number of features, suggests that the IRESpy model shows better generalization. The reduced number of model features results in a decrease in both training time and classification time (making the XGBoost model more appropriate for genome wide scanning).

Surprisingly, incorporation of structural features such as Q_MFE_ and triplet features, has relatively little effect on model performance, although some of the highly ranked features such as “U …” can be directly related to known mechanistic features of some IRES. The reason for this lack of improvement are not obvious. Several explanations seem possible. The extensive nature of the Q_MFE_, while it provides an overall measure of the degree of secondary structure, may not be sensitive enough to particular structural and topological features that are important to IRES function, i.e. a high degree of structure may not be sufficient – specific structures may be required. This seems likely. Alternatively, while the prediction MFE RNA structures is relatively good, generally estimated to be about 80% accurate [32, 51] at the base pair level, it may not be good enough to reliably detect structural motifs. Furthermore, the RNA structure prediction approach used here does not predict pseudoknots which, based our knowledge of viral IRES, may be highly important to IRES function. On the other hand, triplet features take a very local view of structure and sequence, and may be too detailed to capture the important larger structural motifs. Another explanation may be that, in fact, IRES function involves many different mechanisms [37] – the XGBoost decision tree models can capture the fact that different features are important for different IRES, but unfortunately, teasing this information out of the trained model is difficult – the interpretation of the importance of features in machine learning models is a topic of high interest in the machine learning community. The SHAP feature importance plots shown in Fig. 6 can serve as a potential motif list for researchers to test in laboratory experiments. In particular, the triplet “U …” may indicate the importance of a conserved U rich loop motif similar to that found in the SL2.1 region of the Dicistrovirus IGR IRES. The CU kmer is part of a known tetraloop motif (CUYG) which may be important in stabilizing the IRES structure [34]. The combination of global kmer features and structural features increases the validation AUC compared with that of the model incorporating global kmer features alone, but only modestly. Using structural features alone achieves relatively high classification performance, and at the same time, reduces the number of features from 340 to 33. From one point of view, this indicates that the structural features are relatively powerful, providing higher performance per feature, but why these features do not greatly increase predictive performance remains unclear.

## Conclusion

In summary, IRESpy is a high-throughput online tool for IRES prediction. Its prediction quality is better than previous tools, and it is able to predict both viral and cellular IRES with good performance. IRESpy uses only length-independent features in its prediction making in appropriate for analyzing RNAs of different lengths. The computational time is low making IRESpy appropriate for genome wide comparisons and for use in genome annotation. The IRESpy application is freely available as an R/shiny app making it easily available to both computationally sophisticated and more computationally naïve users.

## Methods

### Training data (dataset 2)

We use the same training data as was used for the IRESPredictor model ([10], downloadable at https://bitbucket.org/alexeyg-com/irespredictor/src/v2/data/). This dataset is derived from Weingarten-Gabbay et al. [46] and comprises selected from reported IRES, UTRs of human genes, UTRs of viral genes, and sequences complementary to 18S rRNA. From the original dataset of 55,000 we retain sequences labelled as ‘CDS_screen’, ‘Genome_Wide_Sceen_Elements’, ‘High_Priority_Genes_Blocks’, ‘High_Priority_Viruses_Blocks’, ‘Human_5UTR_Screen’, ‘IRESite_blocks’, ‘Viral_5UTR_Screen’, and ‘rRNA_Matching_5UTRs’ to obtain 28,669 native (non-synthetic) sequences. The removed sequences are mostly synthetic sequences introduced to test the effect of specific mutations on IRES activity. Weingarten-Gabbay et al. screened the sequence fragments in a high-throughput bicistronic assay using a consistent 173 base insert size, removing any length effects. Based on the reported replicate measurements of IRES activity, promotor activity, and splicing activity, we further filtered the dataset to retain only sequences with splicing scores greater than − 2.5 and promoter activity less than 0.2. The final training dataset, referred to as Dataset 2, comprises 20872 subsequences: 2129 sequences with IRES activity scores above 600 are defined as IRES, and the other 18743 as nonIRES. The ratio of IRES to nonIRES is about 1:8.6. This is similar to the ratio of IRES: nonIRES in the human genome, which has been estimated at about 10%.

The similarity of the insert sequences in the 20872 native sequences in Dataset 2 have been checked using Blastn. The results show 7.56% sequences have more than 80% identity, 15.3% sequences have more than 50% identity, and 17.02% sequences have more than 30% identity. There are no sequences with 100% identity. Although the number of high identity sequences is low, the XGBoost model has been retested excluding sequences with higher than 50% identity. We found the model performance is similar (not shown).

### Highly structured RNA data

The highly structured RNA group includes 202 examples of 16S RNA, 23S RNA, 5S RNA, g1 and g2 self-splicing introns, RNaseP, tmRNA and tRNA [13]. The sequences have been carefully screened to remove any sequences with greater than 40% sequence identity.

### Dataset 1

Dataset 1 is composed of sequences from IRESite [33] and selected 5’UTRs of housekeeping genes. Fifty-two viral IRES and 64 cellular IRES from IRESite are labeled as IRES in Dataset 1. Housekeeping genes principally utilize the 5′ cap-dependent mechanism for initiation and 51 of were selected as the non-IRES group in Dataset 1 [24].

### Human UTRs

124315 human 5’UTR sequences were collected from UTRdb [9].

### Kmer features

The frequency of each kmer is calculated as the count of the kmer divided by the sequence length. Global kmer features are counted over the entire length of the sequence. Local kmer features are counted in 20 base windows, with a ten-base overlap between adjacent windows (Fig. 1).

### Predicted minimum free energy (PMFE) and Q_MFE_

The predicted minimum free energy is calculated by UNAfold-3.9 [29].

Q_MFE_ is calculated as follows:

1. Calculate the predicted minimum freedom energy of the secondary structure from the original sequence by RNAfold.
2. The original sequence is randomized while preseerving the dinucleotide frequenciess. Then the MFE of the randomized sequenceis calculated.
3. Step 2 is repeated many times (for example 2000) in order to obtain the distribution of the predicted MFE values.
4. If N is the number of iterations and n is the number of randomized sequences with MFE value less than or equal to the original value, then QMFE is calculated as:

$$ {\mathrm{Q}}_{\mathrm{MFE}}=\frac{\mathrm{n}}{\mathrm{N}+1} $$

The Ushuffle program [18], which is based on the Euler algorithm, is used to randomize the sequences used in calculating the Q_MFE_. Ushuffle uses an exact method that produces randomized sequences with exactly the same dinucleotide composition as the original sequences.

### XGBoost software and parameters

The XGBoost model is fitted under R (Version 3.5.0) with the xgboost package (Version 0.71.2). The parameters used in the XGBoost model include: eta = 0.01, gamma = 0, lamda = 1, alpha = 0, max_depth = 5, min_child_weight = 19, subsample = 0.8, colsample_bytree = 0.65). IRESpy is deployed online as a shiny package (Version 1.2.0). It is available on line: https://irespy.shinyapps.io/IRESpy/.

## Additional file

Additional file 1: Detailed information on 1. nested cross-validation procedure, 2. hyper-parameter tuning, 3. sequence similarity filtering, 4. Performance of VIPS, IRESPred, IRES-Interpreter, and IRESfinder, 5. Genomic scan of Human UTRs for IRES, 6. Feature importance plots, 7. comparison of other ML approaches: Random forest, extremely randomized forest, GLM grid, deep neural net and stacked ensemble models. (DOCX 1300 kb)

## Data Availability

The dataset used to train IRESpy is available online (https://bitbucket.org/alexeyg-com/irespredictor/src).

## Abbreviations

eIFS: Eukaryotic initiation factors
GBDT: Stochastic gradient-boosting decision tree model
IRES: Internal ribosome entry site
ITAFS: IRES trans-acting factors
XGBoost: eXtreme Gradient Boosting
