# Supplementary material for: IRESpy: an XGBoost model for prediction of internal ribosome entry sites
Source: BMC Bioinformatics. 2019 Jul 30;20:409. doi: 10.1186/s12859-019-2999-7 (PMC6664791; doi:10.1186/s12859-019-2999-7)
Supplement: Supplementary file 1 — Detailed information on 1. nested cross-validation procedure, 2. hyper-parameter tuning, 3. sequence similarity filtering, 4. Performance of VIPS, IRESPred, IRES-Interpreter, and IRESfinder, 5. Genomic scan of Human UTRs for IRES, 6. Feature importance plots, 7. comparison of other ML approaches: Random forest, extremely randomized forest, GLM grid, deep neural net and stacked ensemble models. (DOCX 1300 kb) [file 12859_2019_2999_MOESM1_ESM.docx]

Additional file 1:

1. **Nested Cross-Validation**

The model was trained using a nested cross-validation approach, as shown in Fig S1. In the inner loop, 10-fold cross-validation was used to search for the best model with the best hyper-parameters. Since the validation dataset in the outer loop has never been used in the model training, it can be used to test the generalization ability of the model. In the inner loop, ten-fold cross-validation is used to determine the best model with the best hyper-parameters. This model is then applied to predict the validation AUC in the outer loop. The average validation AUC measures the model generalization ability, and the model with the highest validation AUC was picked as the final model.


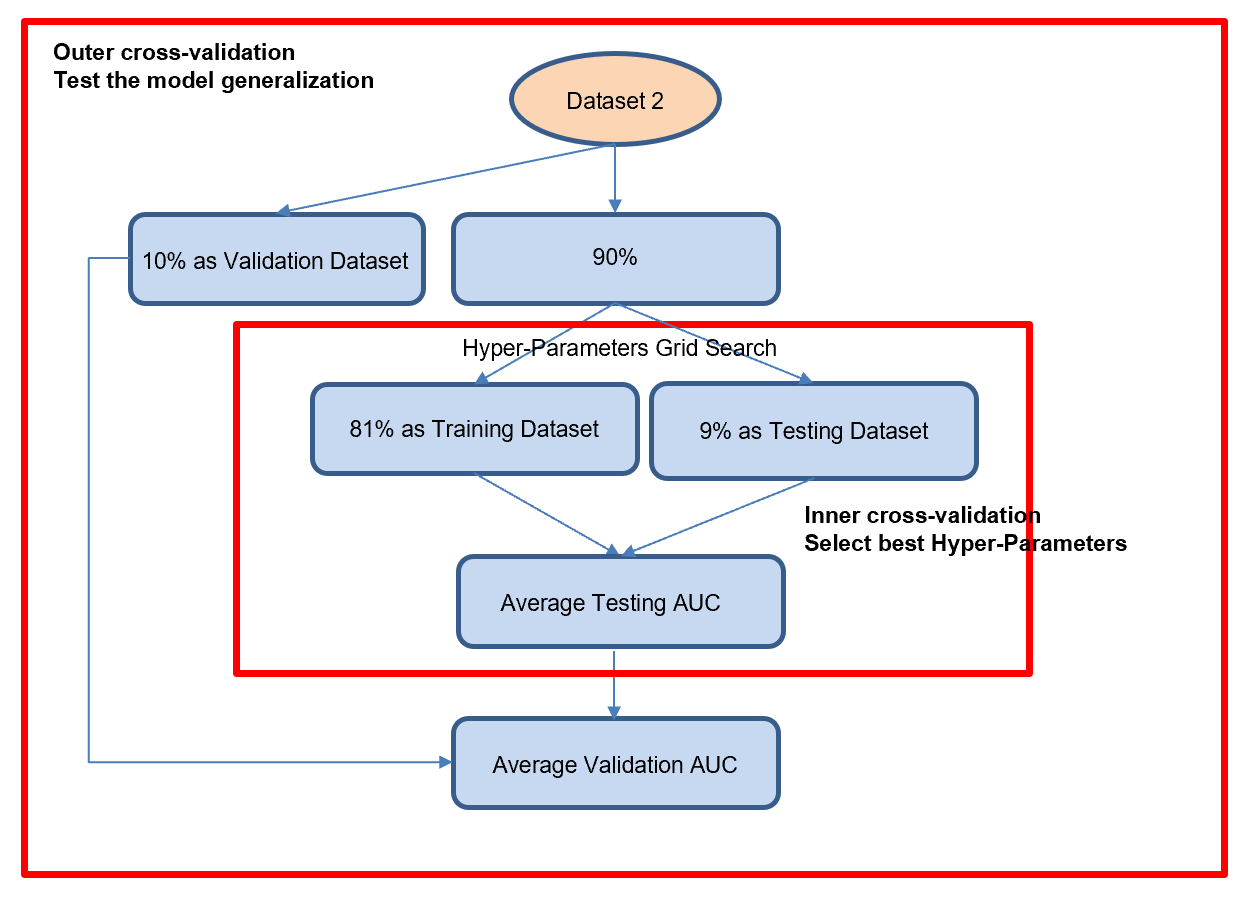


**Fig. S1.** Nested cross validation design map.

1. **Hyper-parameter tuning**

The goal of tuning the hyper-parameters is to obtain the best testing ROC-AUC without over-fitting the training data. There are several important hyper-parameters in XGBoost.

- number of trees
- eta (η): The learning (or shrinkage) parameter, which determines how fast the model converges. The range is 0 to 1.
- max_depth: controls the maximum depth of each tree.
- min_child_weight: controls the minimum number of observations in a leaf.
- colsample_bytree: controls the portion of variables to grow a new node in each tree.
- sub_sample: controls the ratio of the training samples in each tree.
- Gamma (γ): controls the minimum reduction in the loss function required to add a new node to a tree.
- Alpha (α), lambda (λ): L1 and L2 regularization terms on weights.


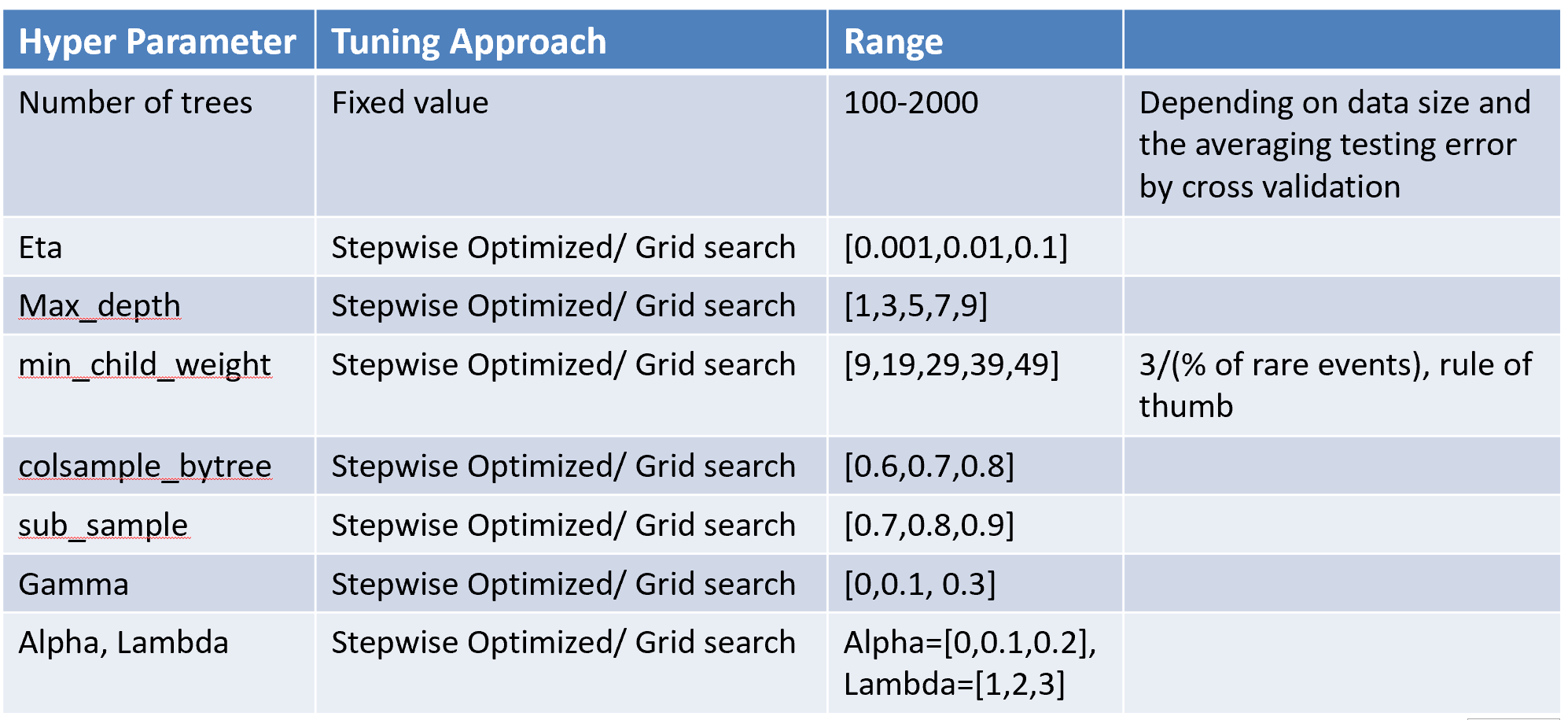


**Table. S1.** Hyper-parameters tune guide.

There are eight hyper-parameters that can be optimized. If all possible combinations of them are tried in a fully grid search, for example, three possible values of eight hyper-parameters, then there are 3^8=6561 different possible combinations. Instead, fixing all parameters except one and optimizing that one is much less time consuming. We first tune the most important hyper-parameters – those which affect the model performance the most. This stepwise optimized search is especially useful and applicable for models with more hyper-parameters to be tuned and more efficient to find a relatively good combination of them. To eliminate some possible bias introduced by that one-by-one tuning, the grid search will be applied on eta, max_depth and min_child_weight in the end.

The suggested parameter ranges for XGBoost models are shown in Table S1 (Chen, 2016). The ranges of the parameters used in the earlier gradient boosting model (Gritsenko et al., 2017), learning rate r=[0.001,0.002,0.004,0.008], minimum leaf samples m=[5,25,125] and subsampling fraction f=[0.9,0.7] have been considered as well. The following approach uses the suggested parameter ranges in Table S1 to tune the hyper-parameters.

My approach for hyper-parameter tuning:

1. Eta, the learning rate, determines how fast to the model is fit. Eta has a huge effect on model performance. Higher eta means faster fitting. Initially, the learning rate is set to a relatively high value (eta=0.01). Then a combination of hyper-parameters within the ranges shown in Table S1 were randomly selected as initial values (showed later). Under these conditions, the optimum number of trees was determined, and the training AUC and testing AUC (Figure S2) calculated.
2. A grid search over the tree-specific hyper-parameters, max_depth, min_child_weight, colsample_bytree, sub_sample and gamma were then performed using the ranges shown in Table S1. The exact tuning process is described below.
3. Next, the regularization parameters, lambda and alpha, were tuned with all the other parameters fixed.
4. The learning rate was tuned with the best combinations of all other hyper-parameters.
5. Grid search of eta, max_depth and min_child_weight to eliminate some possible bias introduced by that one-by-one tuning. The values picked for those parameters are: eta= [0.1,0.01,0.001], max_depth= [1,5,9], min_child_weight= [19,29,39]. So there are 3^3^=27 runs.

I stared with eta=0.01, max_depth=3, min_child_weight=29, colsample_bytree=0.8, sub-sample= 0.8, gamma=0, alpha=0 and lambda=1 as initial values. The training process plot is shown in figure S2-A. It is a good start because the testing AUC approaches a plateau as more trees are trained. The best number of tress is 1661, which is within the suggested range (Table S1). The number of trees is an important hyper-parameter for XGBoost. It usually depends on the size of the training dataset and the range is typical between 100-2000.


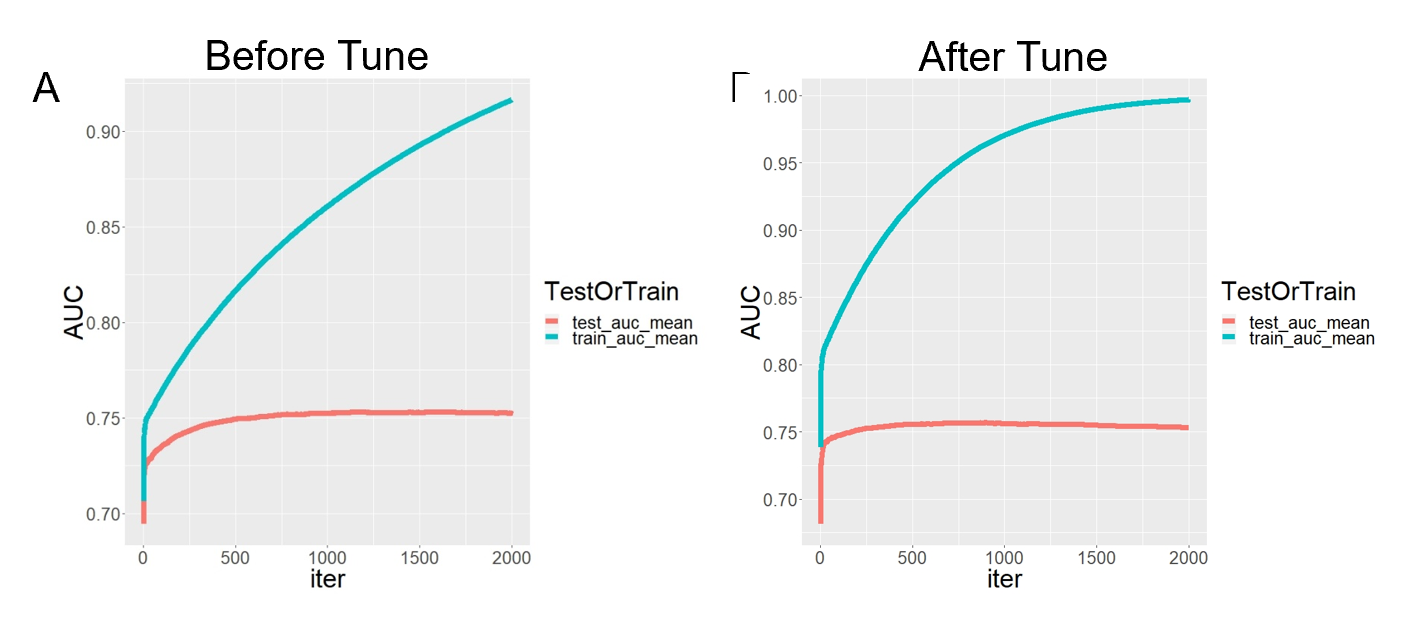


**Fig. S2.** The comparison of Inner loop cross validation performance as trees get bigger before hyper-parameters tuning and after hyper-parameters tuning. Iter, the X-axis, indicates the number of trees. (A). The initial parameters before tuning were: eta=0.01, max_depth=3, min_child_weight=29, colsample_bytree=0.8, sub-sample= 0.8, gamma=0, alpha=0 and lambda=1. The largest test AUC was 0.752, which is obtained when the number of trees is 1661. (B). The parameters after tuning are: eta = 0.01, max_depth=5, min_child_weight=19, subsample=0.8, colsample_bytree=0.7, gamma=0, alpha=0 and lambda=1. The largest test AUC is 0.756 when the number of trees is 901.

After fixing the number of trees at 1661, the other tree-related parameters were tuned in stepwise grid searches. The result is shown in Figure S3. The max_depth and min_child_weight parameters were tuned first because they have higher impact on model performance. The best max_depth is 5 and the best min_child_weight is 19. Then other parameters were tuned one by one later, obtaining gamma=0, subsample=0.8, colsample_bytree=0.7 as the best values.

Alpha is the L1 regularization term on weights and lambda is the L2 regularization term on weights. Increasing either value gives models a higher penalty score for more complexed structures. The grid search shows the default values are the best options for those two hyper-parameters. So alpha=0 and lambda=1.

Finally, eta, the learning rate, was tuned. The results show eta = 0.01 is the best choice. In the end, a grid search of eta, max_depth and min_child_weight has been tried to eliminate some possible bias introduced by that one-by-one tuning. The values picked for those parameters are: eta= [0.1,0.01,0.001], max_depth= [1,5,9], min_child_weight= [19,29,39]. There are 3^3^=27 runs in total and the final results show that eta=0.01, max_depth=5 and min_child_weight=19 is the best combination by getting the highest validation AUC.

Figure S2 shows the change of the model parameters before and after tuning the hyper-parameters. The test AUC has a slightly increase from 0.752 to 0.756. And the number of trees has been decreases from 1661 to 901. It shows the goal of tuning the hyper-parameters which is improving the test AUC but at the same time reduce model complexity.


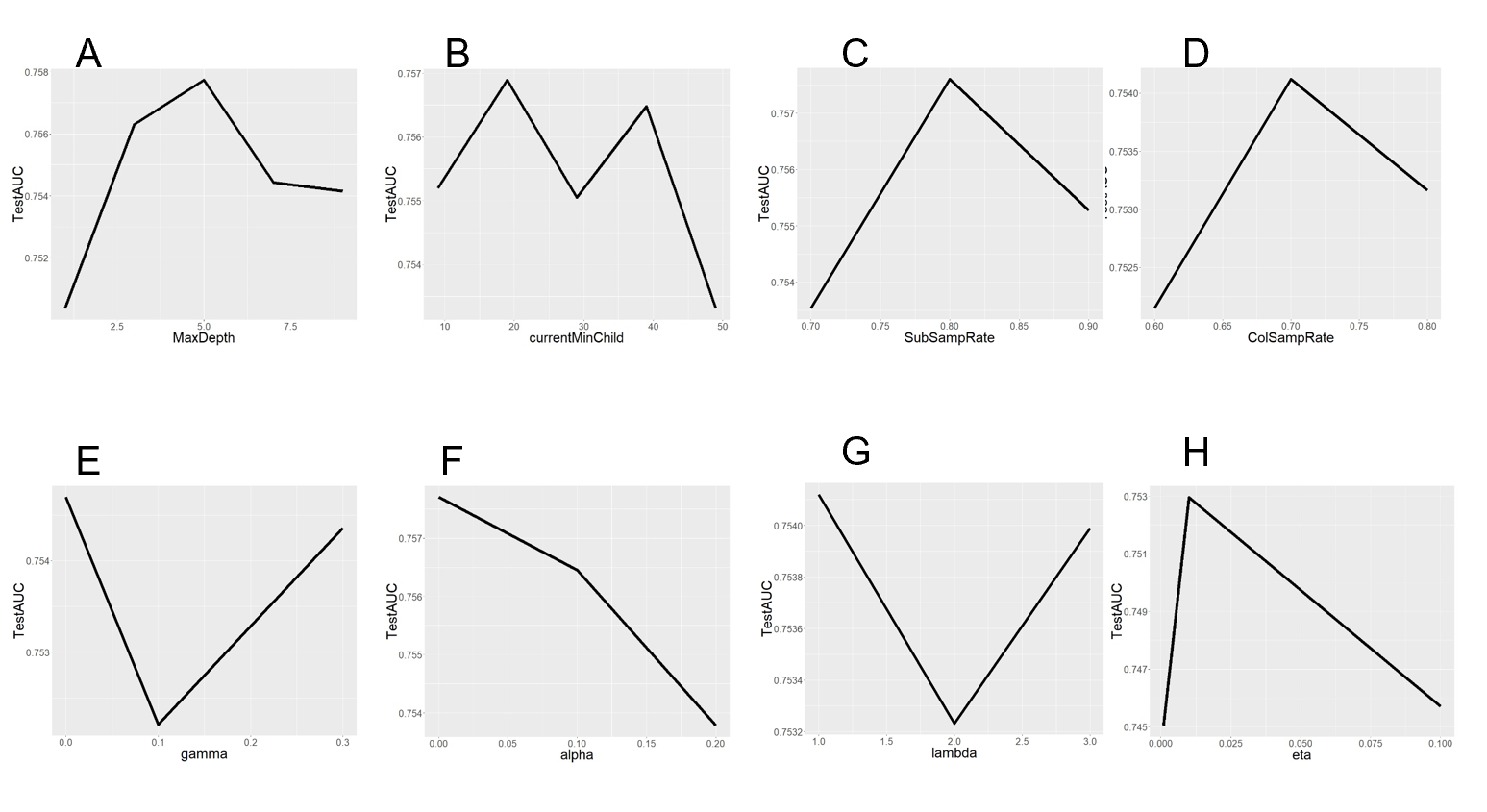


**Fig. S3.** Tree-related hyper-parameter tune results. The effect of varying each parameter separately with the final tuned parameters is shown. (A). Max_depth=[1,3,5,7,9] (B). min_child_weight=[9,19,29,39,49] (C). sub_sample= [0.7,0.8,0.9] (D). colsample_bytree=[0.6,0.7,0.8] (E). gamma=[0,0.1, 0.3] (F). Alpha=[0,0.1,0.2] (G). Lambda=[1,2,3] (A). eta=[0.001,0.01,0.1]

1. **Sequence similarity**

20872 native sequences in Dataset 2 have been checked identity by CD-hit program. CD-hit is a tool for clustering biological sequences on a large scale (Huang, Niu, Gao, Fu, & Li, 2010). It is fast, scalable and flexible based on short word filtering and a greedy incremental clustering algorithm (Li, Jaroszewski, & Godzik, 2002). The results show 7.56% sequences have more than 80% identity, 15.3% sequences have more than 50% identity, and 17.02% sequences have more than 30% identity. There is no any sequence holding 100% identity. When constructing the database, those more than 80% identity sequences are introduced by scanning the similar homologs of viral UTRs, and different sequences sources when scanning the cellular and viral genome. Since the ratio of highly identity sequences is low, the XGBoost model has been tested again by excluding those similar sequences. We found the model performance is similar.


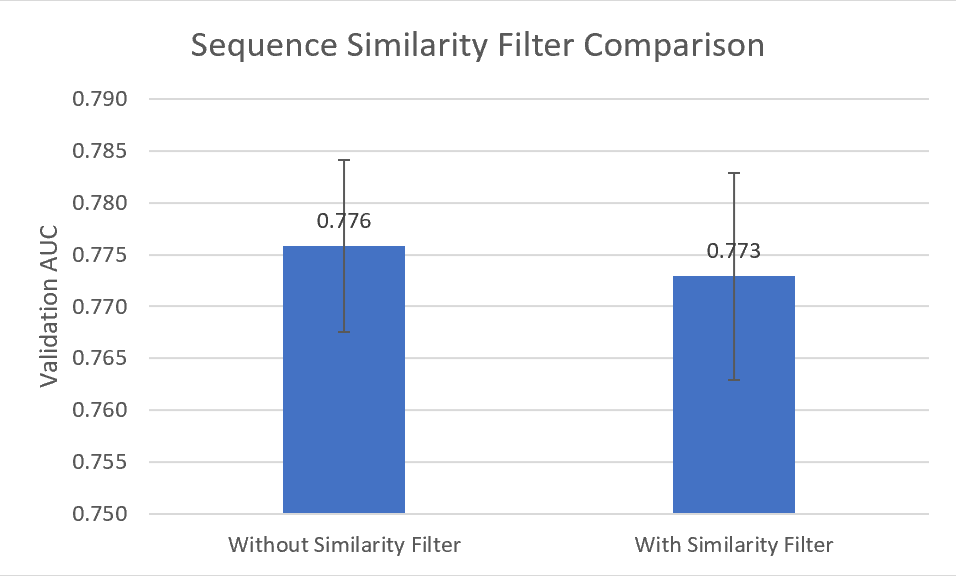


**Fig. S4.** The validation AUC comparison between filtering the 80% sequence similarity and no-filtering the sequence at all.

1. **Model performance comparison**

There are four tools focusing on prediction of IRES before our method IRESpy. Their training dataset, methods, training features, and pros & cons have been listed in Table S2.


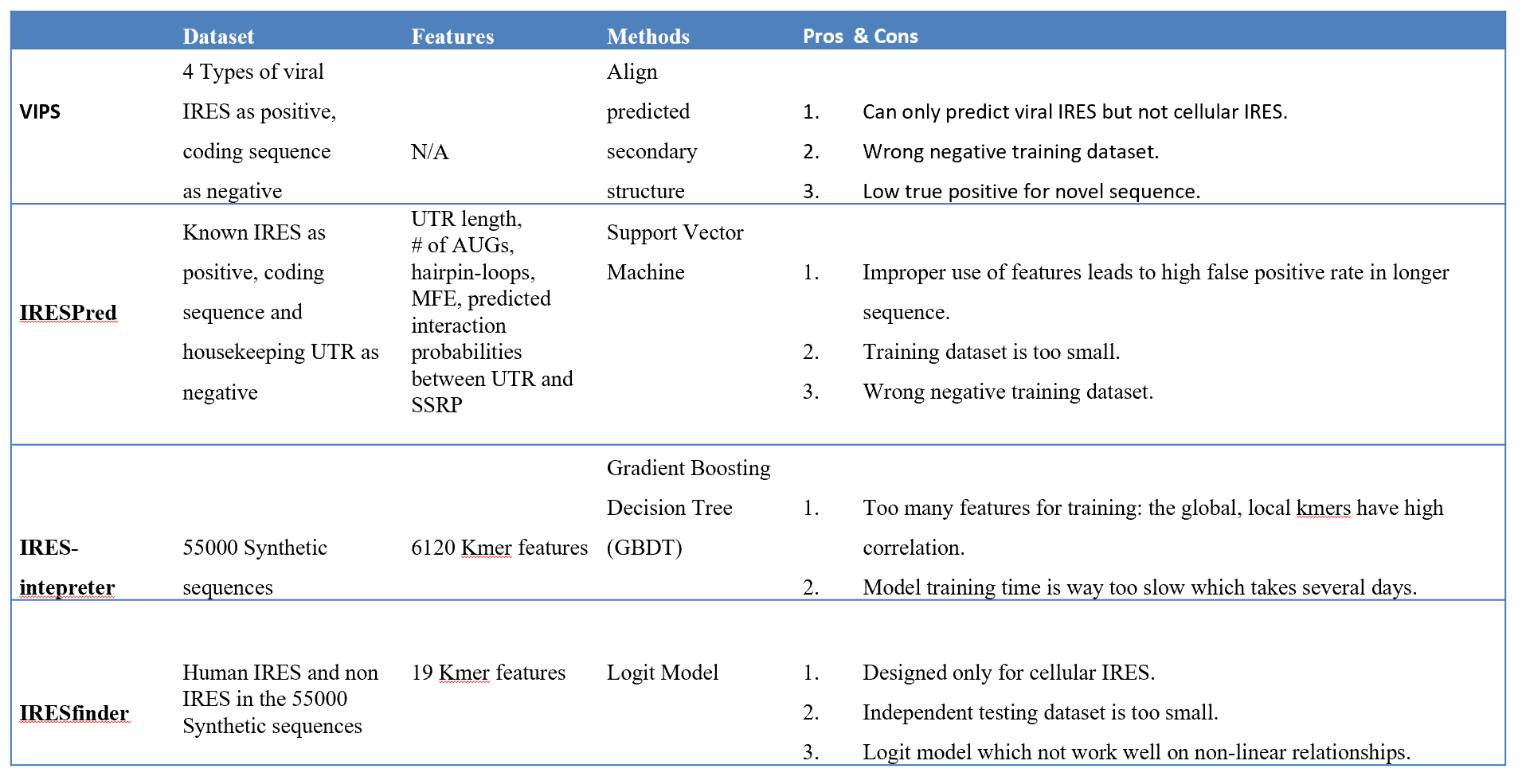


**Table. S2.** Previous IRES prediction model summary.

The summary in Table S2 tells that there are significant defects existing in VIPS and IRESPred. The inclusion of length dependent features such as the length of UTRs, and the number of upstream AUGs in those two methods introduces a serious drawback when predicting IRES in UTRs, which vary greatly in length. VIPS predictions are limited to viral IRES, and although the accuracy rate of VIPS was assessed as over 80% for four viral IRES sub-groups, the prediction accuracy was assessed only on the training dataset and is substantially overestimated. The ability of VIPS to find novel viral IRES is low in our hands (note that the VIPS server is no longer available). IRESpred was trained using a non-IRES negative training set that included viral protein coding and cellular protein coding mRNA sequences; unfortunately some of these sequences were later found to contain IRES (Weingarten-Gabbay et al., 2016). IRESfinder, the most recent method, uses only the human genome part of the Weingarten-Gabbay et al. dataset and implements a logit model with framed kmer features to predict cellular IRES (Zhao et al., 2018). The IRESfinder logit model is trained only on cellular IRES, and, as a transformed linear model, may not work well for non-linear relationships. In addition, the independent testing dataset is very small (only 13 sequences), possibly leading to overestimation of the AUC.

After all, I compare the performance of IRESpy, IRESpred and IRESfinder based on the data from the IRESfinder paper and the result has been shown in Table S3 (Zhao et al., 2018).


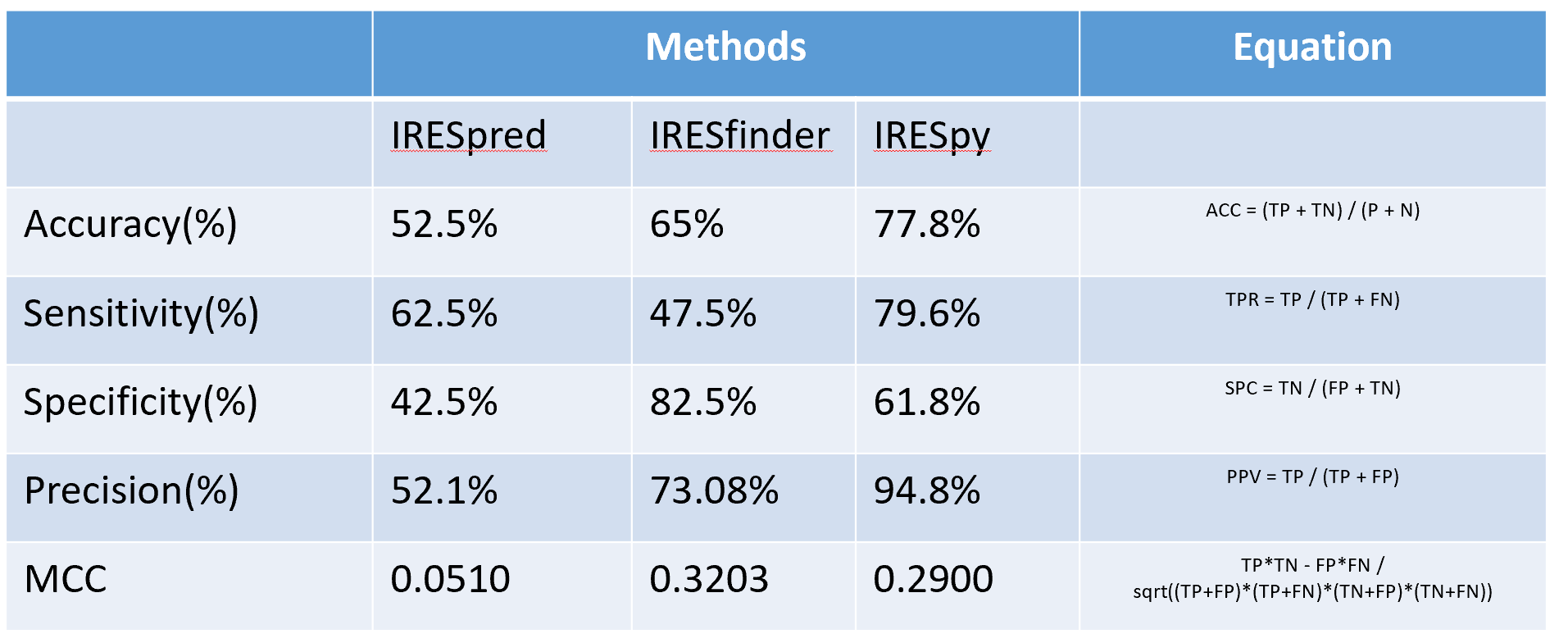


**Table. S3.** Comparison between IRESpy, IRESfinder and IRESpred model performance. IRESpy performs better than IRESpred in accuracy, sensitivity (recall), specificity, precision and MCC.

Even though the defects of IRESpred and IRESfinder exist, IRESpy works better in accuracy, sensitivity and precision in Table S3. The more straightforward comparison is between IRES-interpreter and IRESpy. Because they are working on the same dataset and the algorism of gradient boosting decision tree (GBDT) and the model XGBoost are similar. There is slightly increase of validation AUC from GBDT to XGBoost (Figure 4). But considering the much faster running time to fit the model (Figure 4), IRESpy is a faster, more efficient, more reliable tool to predict IRES compared to IRES-interpreter.

IRESpy provides the first, fast and high-throughput online testing tool for IRES screen. Its website can be used in other genomics applications such as gene annotation and analysis of differential gene expression. The number of features in the model has been greatly reduced, compared to previous predictors, by including global kmer features. It is the first time that structural features such as triplets and QMFE have been explored in the research of IRES. Unlike other structural features like the number of stem-loops and MFE, triplets and QMFE are length independent features and show significance in predicting IRES.

1. **Identification of human 5’UTR IRES**

IRESpy has been applied to scan human 5’UTRs (124315 UTR sequences listed in UTRdb). Fig 9 shows the distribution of IRES prediction probability for the positive and negative training sets in Dataset 2, and all human UTRs. The distribution of probabilities in the human UTR dataset strongly resembles the Dataset 2 negative class, but has a larger tail. This suggests that IRESpy is successfully distinguishing IRES from non-IRES in the uncharacterized human UTRs.


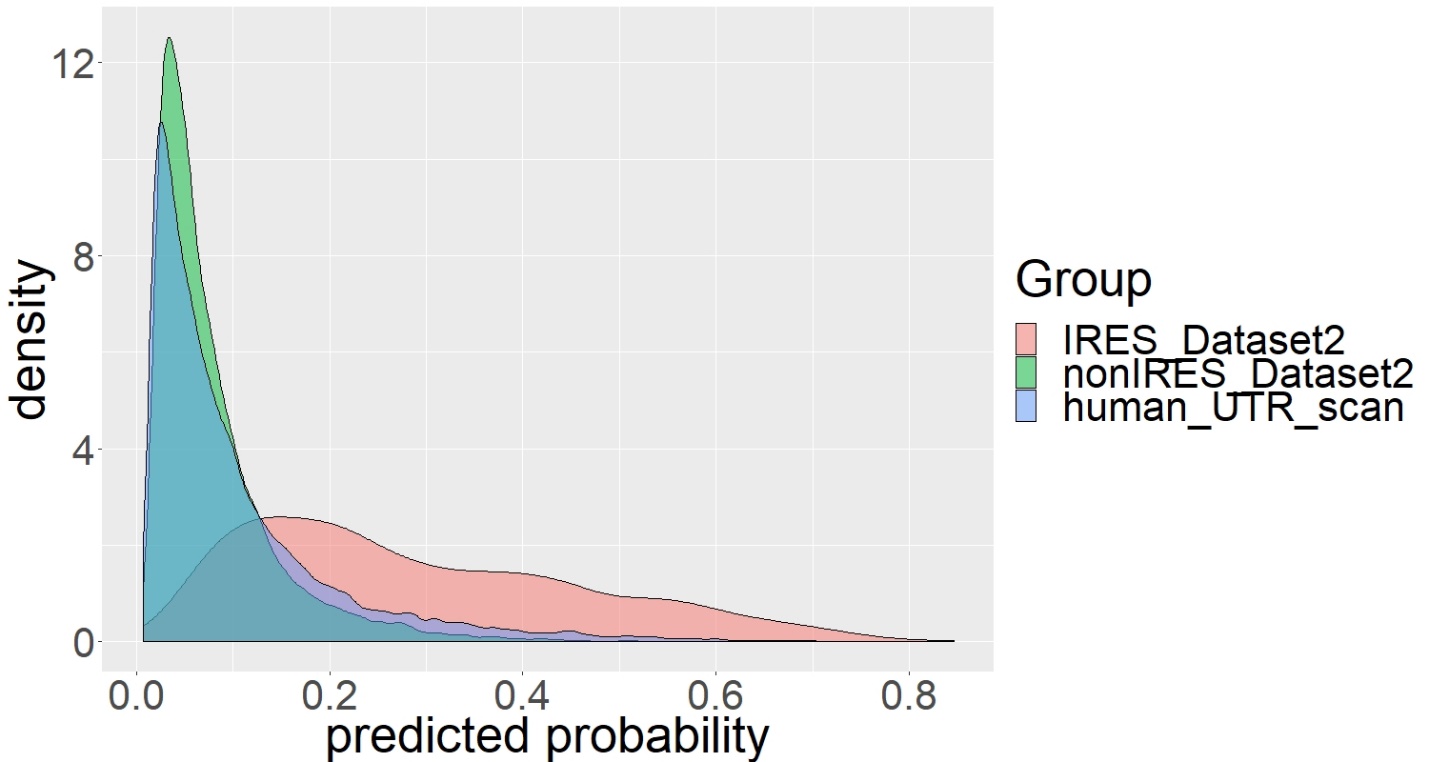


**Fig. 9.** The density distribution of predicted IRES probability in Dataset 2 and human UTR scan.

The top 20 predicted human UTRs by IRESpy has been listed in Table S4. The gene ontology analysis (David 6.8) in Figure S5 shows that IRES might be a widely existing mechanism shared with many biological processes. Spleen tyrosine kinase (SYK), participating in intracellular signal transduction, protein autophosphorylation, Immunity, and protein complex, might potentially utilize IRES. It’s 5’UTR is 201 BP long with a high CT ratio. To future demonstrate the IRES activity, the top 20 predicted UTRs have been aligned by the high-throughput bi-cistronic assay in Weingarten-Gabbay lab (Weingarten-Gabbay et al., 2016). If there is a match, the results have been showed in IRES_activity column in Table S4. The IRES_activity of SYK is 1378.92 compared with a 206 background level. So IRES mechanism might be used by SYK.


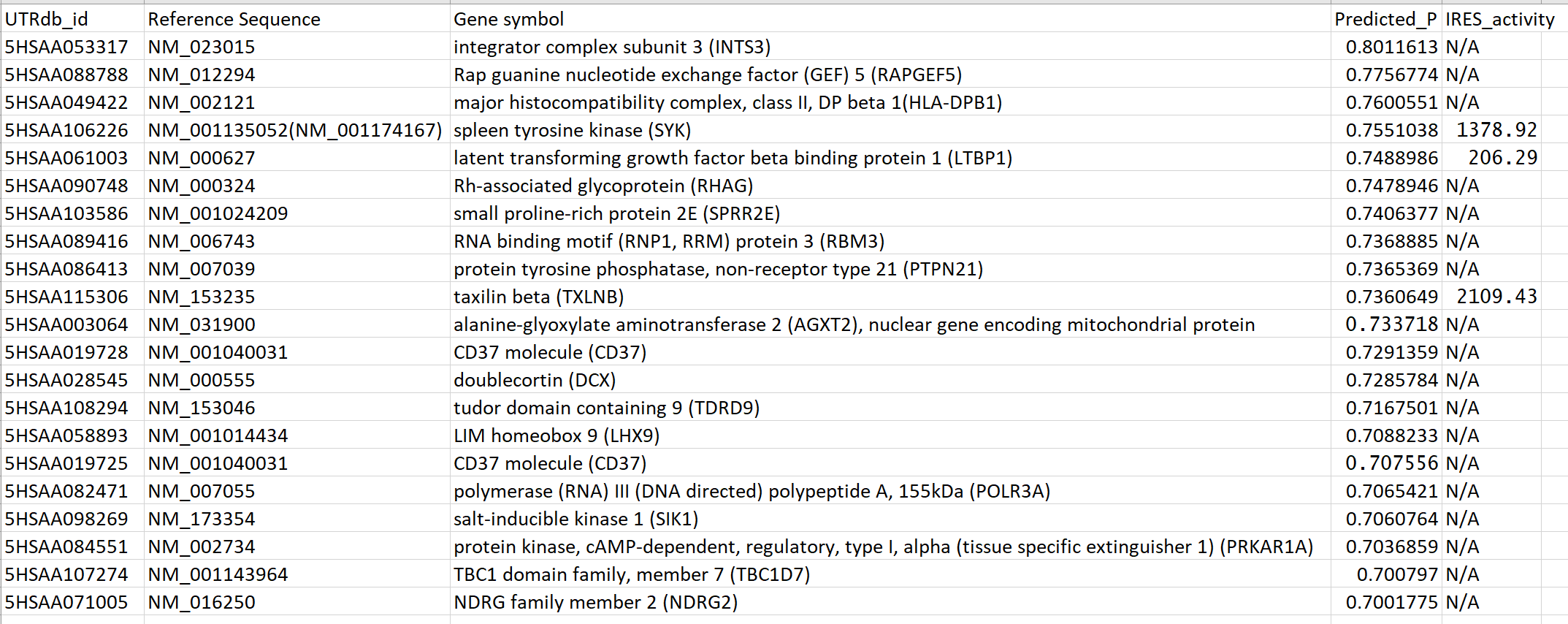


**Table. S4.** Top 20 predicted human UTRs by IRESpy.


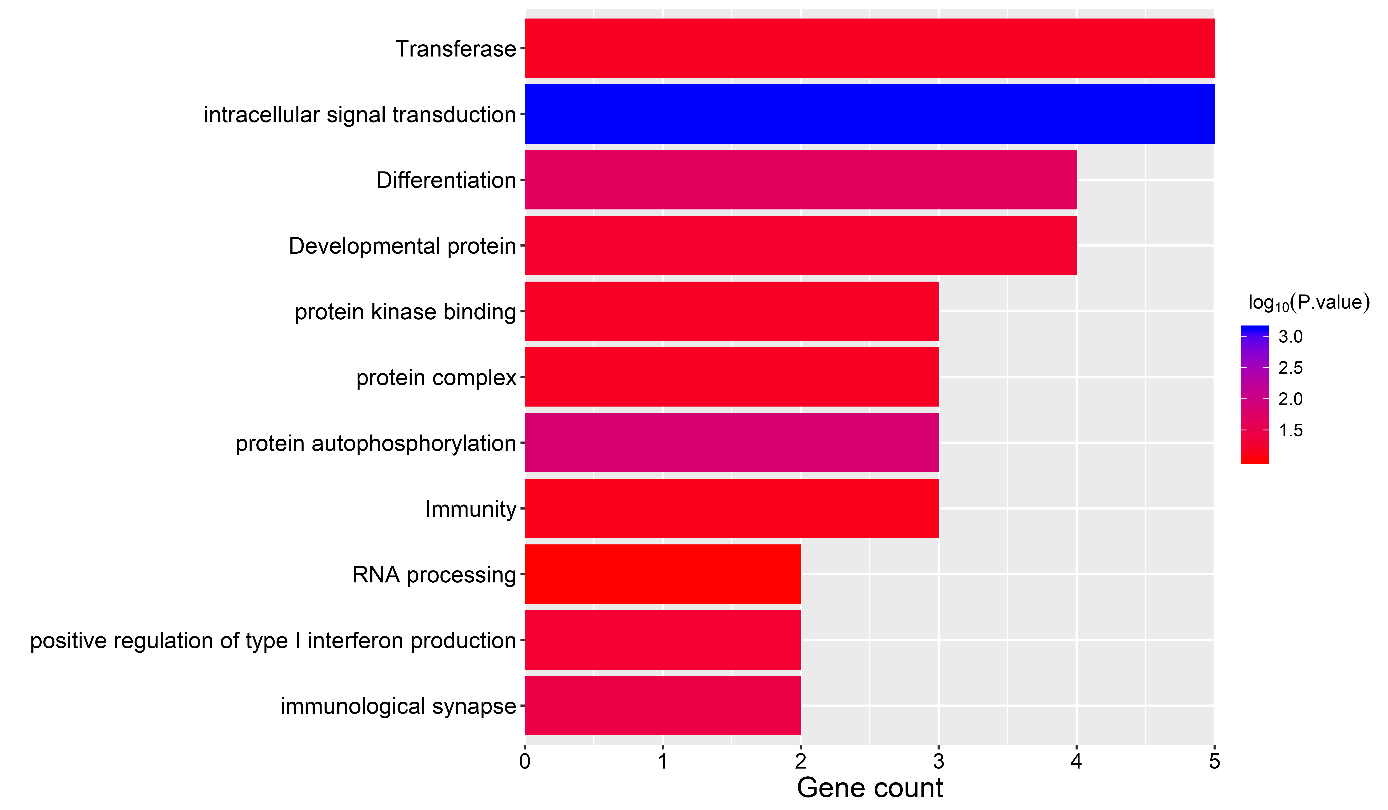


**Fig. S5.** The Gene ontology analysis of the top 20 predicted human UTRs by David 6.8.

1. **The feature importance plot**

As mentioned previously, it is not usually straightforward to understand the biological relevance of the selected features. Machine learning (ML) models are often considered “black boxes” due to their complex inner mechanism. Understanding the contribution of each feature to the model has been recognized as a very difficult aspect of machine learning. The SHAP (SHapley Additive exPlanations) method assigns values that measure the marginal contribution of each feature in the model (Lundberg, 2017). It combines game theory with local explanations and is well suited for machine learning explanation. Unlike feature importance measures based on weight, cover, or information gain, the SHAP value is the only consistent and locally accurate additive method, and it can be interpreted as indicating which features are the most globally important for classification. Figure 6A shows the top 20 most important features in models trained with both global and local kmers. Red indicates higher feature values and blue indicates lower feature values. Higher frequencies of U rich kmers, such as “U”, “UU”, “UUU”, “UUUU”, “CU”, and “UGU”, are associated with higher predicted probability of being IRES. This is consistent with the previous reports that pyrimidine-rich kmers, especially U rich kmers are important for IRES function. (Weingarten-Gabbay et al., 2016). Those U rich kmers make the single nucleotide “U” significantly pushing the prediction to be more likely to be IRES. Importance of global kmer and local kmer features follow similar patterns, for instance, the local kmer features U_121, U_131, U_141, U_151, and U_161 all support classification of sequences as IRES, as do the global kmer features. The importance of the local region from base 121-161 may be important as an ITAF binding site (perhaps pyrimidine tract binding protein), as suggested by Weingarten-Gabbay et al. Whether the CU feature is related to the poly U feature is difficult to tell. It is worth noting that in picornoviral IRES, one of the most conserved features is the SL3A “hexaloop” in which a CU dinucleotide is highly conserved (Fernandez, Buddrus, Pineiro, & Martinez-Salas, 2013). Figure 6B lists the SHAP values of the top important features for the global kmer only model. The similar importance of features in different models suggests that the models are detecting essentially the same features. Figure 6C shows the SHAP values for both the global kmer and structural features model. Some structural features, such as ‘U..’, ‘G(((’, and the QMFE , are more important than most global kmers. Figure 6D lists the structural features which serves as a potential structural motif list much like a differentially expressed genes list in the RNA-seq analysis.


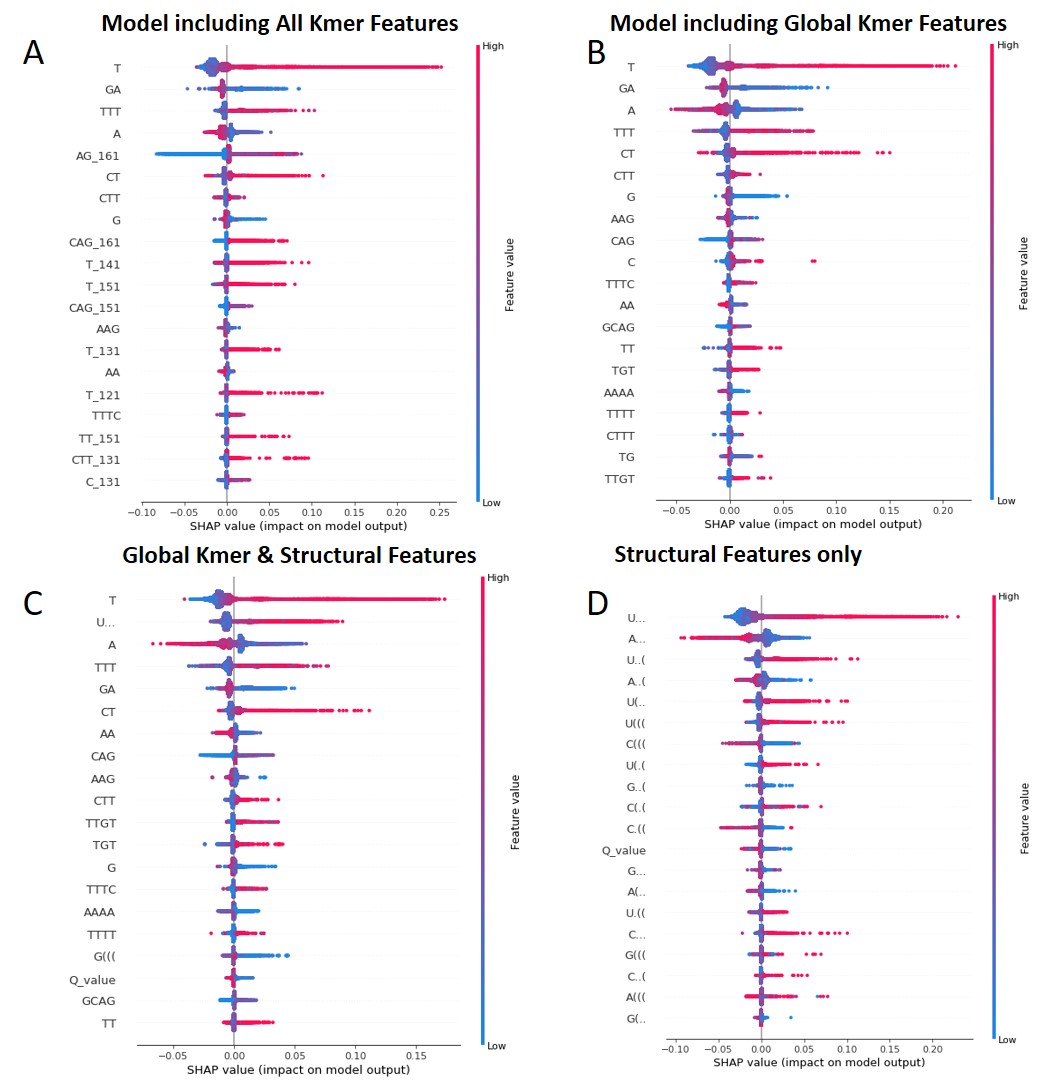


**Fig. 6.** XGBoost model feature importance explained by SHAP values at the global scale. A. The summary of SHAP values of the top 20 important features for model including both global kmers and local kmers. B. The summary of SHAP values of the top 20 important features for models including only global kmers. C. The summary of SHAP values of the top 20 important features for models including both global kmers and structural features. D. The summary of SHAP value of the top 20 important features for model including only structural features.

Chen, T., & Guestrin, C. (2016). Xgboost: A scalable tree boosting system. *In Proceedings of the 22nd acm sigkdd international conference on knowledge discovery and data mining, pp. 785-794*.

Fernandez, N., Buddrus, L., Pineiro, D., & Martinez-Salas, E. (2013). Evolutionary conserved motifs constrain the RNA structure organization of picornavirus IRES. *FEBS Lett, 587*(9), 1353-1358. doi:10.1016/j.febslet.2013.03.005

Gritsenko, A. A., Weingarten-Gabbay, S., Elias-Kirma, S., Nir, R., de Ridder, D., & Segal, E. (2017). Sequence features of viral and human Internal Ribosome Entry Sites predictive of their activity. *PLoS Comput Biol, 13*(9), e1005734. doi:10.1371/journal.pcbi.1005734

Huang, Y., Niu, B., Gao, Y., Fu, L., & Li, W. (2010). CD-HIT Suite: a web server for clustering and comparing biological sequences. *Bioinformatics, 26*(5), 680-682. doi:10.1093/bioinformatics/btq003

Li, W., Jaroszewski, L., & Godzik, A. (2002). Tolerating some redundancy significantly speeds up clustering of large protein databases. *Bioinformatics, 18*(1), 77-82.

Lundberg, S. M., & Lee, S. I. (2017). A Unified Approach to Interpreting Model Predictions. *In Advances in Neural Information Processing Systems*.

Weingarten-Gabbay, S., Elias-Kirma, S., Nir, R., Gritsenko, A. A., Stern-Ginossar, N., Yakhini, Z., . . . Segal, E. (2016). Comparative genetics. Systematic discovery of cap-independent translation sequences in human and viral genomes. *Science, 351*(6270). doi:10.1126/science.aad4939

Zhao, J., Wu, J., Xu, T., Yang, Q., He, J., & Song, X. (2018). IRESfinder: Identifying RNA internal ribosome entry site in eukaryotic cell using framed k-mer features. *J Genet Genomics*. doi:10.1016/j.jgg.2018.07.006
